# Phase of firing does not reflect temporal order in sequence memory of humans and recurrent neural networks

**DOI:** 10.1038/s41593-025-01893-7

**Published:** 2025-03-24

**Authors:** Stefanie Liebe, Johannes Niediek, Matthijs Pals, Thomas P. Reber, Jennifer Faber, Jan Boström, Christian E. Elger, Jakob H. Macke, Florian Mormann

**Affiliations:** 1 Department of Epileptology, University of Bonn Medical Center, Bonn, Germany; 2 University Hospital Tübingen, Department of Neurology and Epileptology, Tübingen, Germany; 3 Machine Learning Group, Technische Universität Berlin, Berlin, Germany; 4 Machine Learning in Science, Excellence Cluster Machine Learning, Tübingen University, Tübingen, Germany; 5 Tübingen AI Center, Tübingen, Germany; 6 Faculty of Psychology, UniDistance Suisse, Brig, Switzerland; 7 Department of Neurology, University of Bonn Medical Center, Bonn, Germany; 8 Department of Neurosurgery, University of Bonn Medical Center, Bonn, Germany; 9 Max Planck Institute for Intelligent Systems, Tübingen, Germany

**Keywords:** Cognitive neuroscience, Network models, Working memory

## Abstract

The temporal order of a sequence of events has been thought to be reflected in the ordered firing of neurons at different phases of theta oscillations. Here we assess this by measuring single neuron activity (1,420 neurons) and local field potentials (921 channels) in the medial temporal lobe of 16 patients with epilepsy performing a working-memory task for temporal order. During memory maintenance, we observe theta oscillations, preferential firing of single neurons to theta phase and a close relationship between phase of firing and item position. However, the firing order did not match item order. Training recurrent neural networks to perform an analogous task, we also show the generation of theta oscillations, theta phase-dependent firing related to item position and, again, no match between firing and item order. Rather, our results suggest a mechanistic link between phase order, stimulus timing and oscillation frequency. In both biological and artificial neural networks, we provide evidence supporting the role of phase of firing in working-memory processing.

## Abstract (web summary)

The temporal order of events in working memory is thought to be reflected by ordered neuronal firing at different phases. Here the authors show that this is not the case and that phase order is linked to stimulus timing and oscillation frequency.

## Main

How do we maintain the temporal order of a sequence of events in memory? Performing this kind of task is an integral part of our ability to encode, maintain and retrieve memories within their spatial and temporal context. The medial temporal lobe (MTL) has been heavily implicated in memory processing at the neural level. For example, hippocampal neurons exhibit elevated, stimulus-specific spiking activity during the maintenance period of memory tasks^1–3^. Another hallmark neural signature of the MTL are oscillations in the frequency range of 2–8 Hz, commonly known as the theta band. Theta oscillations can be measured from local field potential (LFP) or intracortical electroencephalogram/electrocorticogram and have been ubiquitously observed in many species during memory processing^4,5^. Specifically, the amount of oscillatory activity, that is, theta power, increases during memory maintenance and correlates with memory load and task performance^6^.

Combined measurements of spiking and LFPs from MTL have further established an important link between single-neuron firing and theta oscillations: the firing of MTL neurons depends on theta phase—so-called spike-phase coupling—and phase of firing contains information about multiple spatial locations during sequential spatial encoding as well as spatial memory tasks in rodents^4,7,8^.

In analogy to spatial memory in rodents, it has been suggested that this so-called temporal code is also suitable to represent multi-item sequences during working memory of nonspatial information. Specifically, a prominent computational model by Lisman and colleagues hypothesizes that the order of items held within memory is represented by spiking of sequentially reactivated neurons at different phases of theta oscillations^9^.

Indeed, human MTL neurons show preferential firing with respect to theta phase in memory tasks^10^, the magnitude of spike-phase coupling is predictive of subsequent memory performance^11^ and spiking relative to theta phase contains nonspatial information, namely stimulus identity^3,12^. However, thus far it remains unclear how memorizing the sequential order of multiple items is implemented at the neural level in the human MTL. In particular, it is unknown whether a sequence of memorized items is associated with (1) differences in theta-related phase of firing of single neurons and (2) whether the order of phase of firing matches the item order, as hypothesized by Lisman’s theory^9^.

Here, we sought to answer these questions by directly measuring both spiking of MTL neurons and LFPs while participants had to maintain the temporal order of a sequence of items in working memory. We also investigated potential underlying neural mechanisms by training recurrent neural networks (RNNs) to perform an analogous task, without explicitly instructing RNNs on how to solve it. Our results show emerging theta oscillations and spike-phase coupling in both recorded and modeled neural activity during working memory, where phase of firing is related to item position within a sequence. Surprisingly, however, phase order did not match item order, in contrast to Lisman’s theory. Instead, our modeling suggests that phase order could arise as a function of stimulus onset asynchrony and oscillation frequency—a relationship we subsequently corroborated in our neural recordings. Our findings thus validate, but also challenge, a long-standing theory about the role of spiking and oscillations in memory function.

## Results

### No effect of item position on spike rate during working memory

**Fig. 1:**
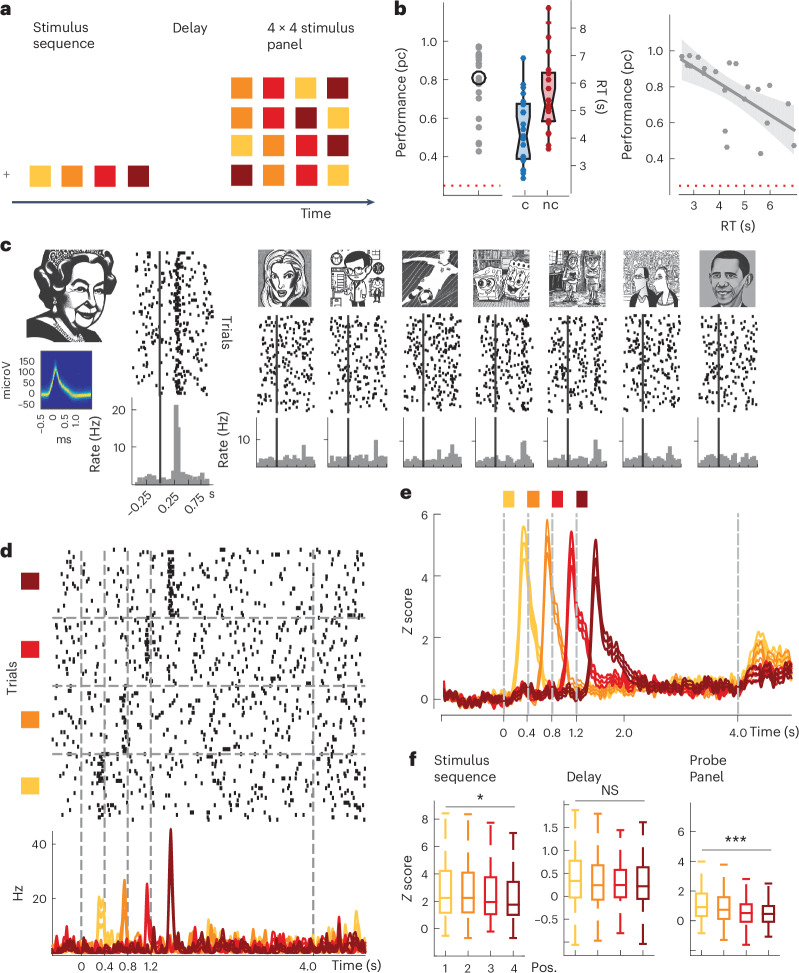
Experimental paradigm, behavioral performance and spike rate modulation. **a**, The experimental design of the multi-item temporal-order working-memory task. **b**, Left: individual mean percentage correct (pc) per session (mean pc 77%, s.d. 17,8%) for each patient and session (*N* data points correspond to 21 sessions, from 16 patients) compared with chance performance at 25% (dashed red line). Binomial test, *P* = 2.8 × 10^−61^, one sided. The black circle shows the average across subjects and median RTs for correct (c) versus incorrect (nc) trials, box plots are centered at the median and depict the interquartile range; whiskers represent 1.5 times the interquartile range, that is, a Tukey box plot. Right: median RTs plotted against mean percentage correct with least-squares linear fit and s.e.m. show a negative correlation (Spearman rank correlation coefficient −0.69, *P* = 6.7 × 10^−4^ two sided, median RT 4,514 ms, s.d. 1,328 ms). **c**, Spike waveform density plot (microV, microVolt), raster plots and peristimulus time histograms (PSTH) from a hippocampal unit to all stimuli shown in one session. The vertical lines correspond to stimulus onset. This unit showed a significant rate increase to only one of the eight stimuli (PS, one-sided Wilcoxon signed-rank test, *P* = 4.3 × 10^−13^). **d**, Spiking activity of the same unit across the entire trial period in response to the PS shown at four different positions within the sequence (one-way repeated measures analysis of variance, *F* = 3.05, *P* = 0.03 during the stimulus period). **e**, Convolved peristimulus time histogram and s.e.m. (*Z* score relative to baseline) in response to the PS of each individual neuron, averaged across all stimulus-responsive units for the entire trial period (sequence position color coded). **f**, Median spike rates for each stimulus position (pos.) and specific trial periods. The box plots are centered at the median and depict interquartile range, whiskers represent 1.5 times the interquartile range (Tukey box plot, Kruskal–Wallis nonparametric analysis of variance, on stimulus-evoked response, chi-squared of 7.96,*P* = 0.04, probe panel chi-squared of 39.5, *P* = 1.35 × 10^−8^, delay chi-squared of 2.55, *P* > 0.05, *N* = 217 visually responsive neurons). Note that the stimuli used in the experiments cannot be displayed for copyright reasons and have been replaced by thumbnails generated with stable diffusion (https://huggingface.co/spaces/stabilityai/stable-diffusion).

We recorded spiking activity of 1,420 units and LFPs from 921 channels in MTL regions including the hippocampus (HPC), entorhinal cortex (EC), parahippocampal cortex (PHC) and amygdala (AM) in 16 patients with epilepsy undergoing presurgical seizure monitoring. Patients performed a sequential multi-item working-memory task (Fig. 1a). After a fixation period, four randomly chosen pictures out of a set of eight were sequentially presented to the patients for 200 ms each (400 ms between stimulus onsets). After this sequence and a subsequent delay period of 2,500 ms (±100 ms), a panel comprising four rows of picture sequences appeared, one of them matching the previously presented sequence. Patients indicated a match by pressing a key on a keyboard. Each patient performed markedly above chance but in a range that allowed us to compare neural activity for correct versus incorrect trials. Median reaction time (RT) showed a negative correlation with mean performance across subjects (Fig. 1b). In summary, our behavioral results indicate that subjects understood the task well and were generally attentive during participation.

While spike rates of MTL units are typically modulated by stimulus identity, little is known about whether the sequential position of stimuli systematically affects spiking. To address this question, we identified 217 highly responsive units by comparing stimulus-evoked activity during encoding to a prestimulus baseline (HPC: *N* = 84; EC: *N* = 23; PHC: *N* = 55; AM: *N* = 55; Methods). An example response of such a unit is shown in Fig. 1c. The stimulus-evoked activity of this unit differed substantially in response to the PS at each of the four positions within the sequence (Fig. 1d) during encoding, with the largest evoked response at the end position of the sequence and no modulation during delay or the probe period. Assessing spiking across all stimulus-responsive units for the PS at each item position, however, we found that stimulus-evoked responses were typically largest for the first position and appeared to systematically decrease with increasing sequence position (Fig. 1e). Interestingly, we observed a similar effect during the panel presentation. Here, spiking activity was largest whenever the units’ PS had been shown as the first within the initial sequence, even though a 4 × 4 picture grid was displayed (Fig. 1f). In contrast, during the delay period, spike rates did not differ between stimulus positions, although spiking was significantly elevated during the delay compared with the prestimulus baseline, suggesting maintenance-related activity (see also ref. ^1^), (Wilcoxon signed-rank-test, *Z* > 2.7, *P* < 0.001 for each region). To investigate whether stimulus position could be decoded from firing rates during the three phases of each trial, we used a support vector machine (SVM) algorithm. Position could be decoded during the encoding and retrieval phases but not during delay (permutation test against shuffled position labels, *P* < 0.05 for sample/probe, *P* > 0.05 for delay, *N* = 199 shuffles; Supplementary Fig. 1).

In summary, MTL neurons show robust stimulus-specific encoding responses during visual presentation. During encoding, decreased spiking with increased item position might be related to the so-called primacy effect that has been observed in serial memory for mesoscopic brain signals^13^. In contrast, no such differences were observed during memory. Thus, the remembered item order does not seem to be reflected in systematic spike-rate changes in the MTL.

### Theta oscillations and spike-phase coupling during memory

**Fig. 2:**
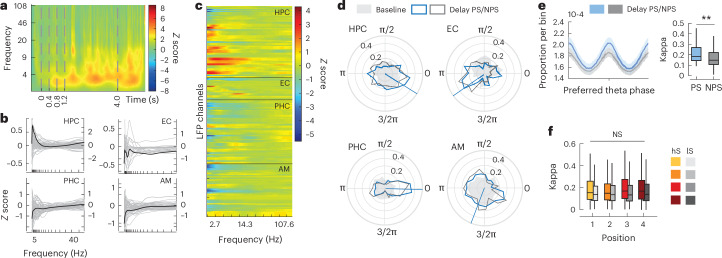
Theta oscillation and spike-phase modulation during sequence maintenance. **a**, An example normalized time–frequency spectrum (power *Z* score relative to baseline) in anterior HPC (the same recording site as in Fig. 1) showing a sustained increase in theta power during the delay. **b**, Normalized power spectra during the delay per MTL region (black median, single gray channels). **c**, The same as **b**, but color coded for each channel. **d**, Preferred phase distribution in the theta range for all stimulus-responsive units during baseline (shaded) and delay where sequences contained the PS (blue) or NPS (gray). The lines show the mean preferred phase angle during delay. **e**, Left: mean (95% confidence intervals) spike-phase histogram centered at each individual unit’s preferred phase during the delay, separately for PS versus NPS trials. Right: median estimated concentration parameters for PS versus NPS trials based on individual von Mises fits, Tukey box plots (two-sided Wilcoxon signed-rank test, *N* = 217, *Z* = 8.85, *P* = 8.9 × 10^−19^). **f**, Kappa separately per stimulus position for more selective (hS, high-selectivity; yellow–red) versus less selective units (iS, low-selectivity; light–dark gray). Two-sided Kruskal–Wallis test based on individual values, *P* > 0.05, chi-squared of <3.9.

Previous studies have observed increases in theta oscillations, that is, power (2–8 Hz) during working-memory maintenance^6^. Thus, we first asked whether we find similar enhancements in our task. An example time–frequency spectrogram recorded from the hippocampal site shown in Fig. 1 is plotted in Fig. 2a. A clear increase in theta power around 2.8 Hz throughout the delay is visible (median power baseline versus delay, Wilcoxon signed-rank test, *P* < 0.01). We generally observed a similar effect comparing power across all LFP channels carrying stimulus-responsive units between baseline and delay (Wilcoxon signed-rank test, *N* = 217, *Z* = 3.7, *P* < 0.001; Supplementary Fig. 2a,b), which was most prominent in the HPC and EC (the proportion of channels exhibiting elevated theta during maintenance: HPC: 40%; EC: 48%; PHC: 20%; AM: 36%, binomial test, *P* < 1 × 10^−4^ at *α* = 0.01 for every region). Thus, our results confirm earlier findings on memory-related theta power increases during working memory and provide the basis for our following analyses.

Combining single-unit and LFP recordings, we next analyzed spiking as a function of theta phase (Fig. 2d). Spike-phase angles across the neuronal population showed a nonuniform distribution during the delay for all MTL regions (Rayleigh’s test, *Z* > 6.1, *P* < 0.01 for all four MTL regions), whereas this was only the case for hippocampal and AM neurons during prestimulus baseline (HPC: *Z* = 4.63; AM: *Z* = 4.09; both *P* < 0.05; PHC: *Z* = 3.21; EC: *Z* = 2.94; *P* > 0.05, after correcting for possible spike-rate differences between baseline and delay). Median phase angles also differed considerably between MTL regions during the delay. This was not the case during baseline at the population level (nonparametric multisample test comparing median angles between regions *P* > 46.2 and *P* < 10^−10^ for delay PS or not (NPS) and *P* = 7.63 and *P* > 0.05 for baseline, respectively), neither comparing spike-phase locking magnitude between individual units (median comparison Rayleigh-based *Z* scores on individual units, Wilcoxon signed-rank test, *Z* > 5.9, *P* < 1 × 10^−9^ or kappa *Z* > 2.7, *P* < 1 × 10^−3^ for all regions, see also Supplementary Fig. 2c). We also observed that theta modulation of spiking was elevated during maintenance if the PS was encoded in the stimulus sequence versus not (NPS trials; Fig. 2e).). This indicates that increased theta spike coupling is associated with memorizing specific stimulus information (Wilcoxon signed-rank test comparing median values based on individual unit’s von Mises fits, *N* = 217, *Z* = 8.65, *P* < 0.001). In contrast, the strength of spike-phase coupling did not depend on serial stimulus position (Kruskal–Wallis test based on individual values, *P* > 0.05, chi-squared of <3.9; Fig. 2f). Taken together, memory maintenance was associated with increased theta spike-phase locking, where phase-of-firing distributions became less uniform as units showed more similar phase preferences during the delay. For individual units, increased spike-phase coupling was specific to the encoded stimulus. Interestingly, similar to our spike-rate analyses, the magnitude of phase coupling was not systematically related to the position of the stimulus within a sequence.

### Phase of firing depends on sequence position

**Fig. 3:**
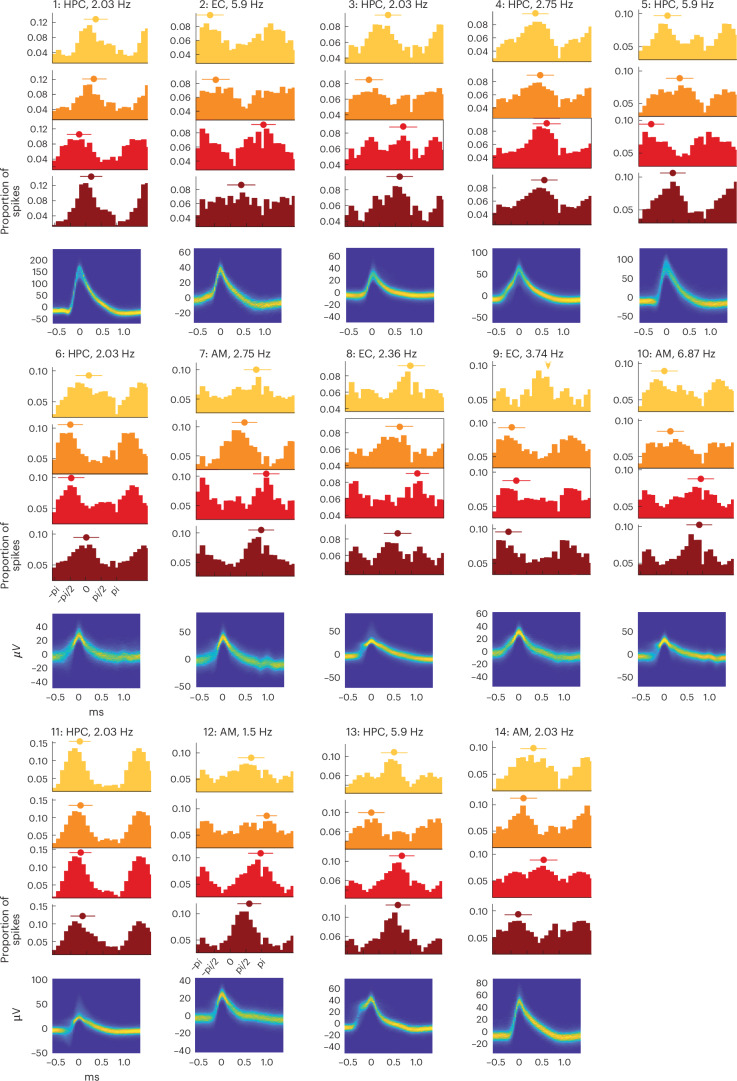
Phase of firing in single neurons encodes sequence position. Spike waveforms (bottom row) and spike-phase histograms (upper three rows) of single units derived at different stimulus positions (color coded). Mean preferred phase and circular standard deviation are shown above the histograms in each plot, as well as the theta frequency. Units are sorted in ascending order based on the *P* value obtained from comparing *V*_ex_ permutation tests (two sided); all units show *P* < 0.05.

We next focused on the temporal relationship between theta oscillations and spiking. We asked whether the preferred phase of firing during the delay varies with item position in a sequence, one of the central model predictions by Lisman and Idiart^9^. Analyzing spike-phase histograms, we found differences in single units’ preferred phase of firing depending on the sequence position of the PS (Fig. 3). We quantified this relationship by computing the circular variance explained (*V*_ex_; Methods) between phases at different sequence positions. To assess statistical significance for each unit, we compared *V*_ex_ with a null distribution derived from randomly shuffling position labels (Fig. 3).

**Fig. 4:**
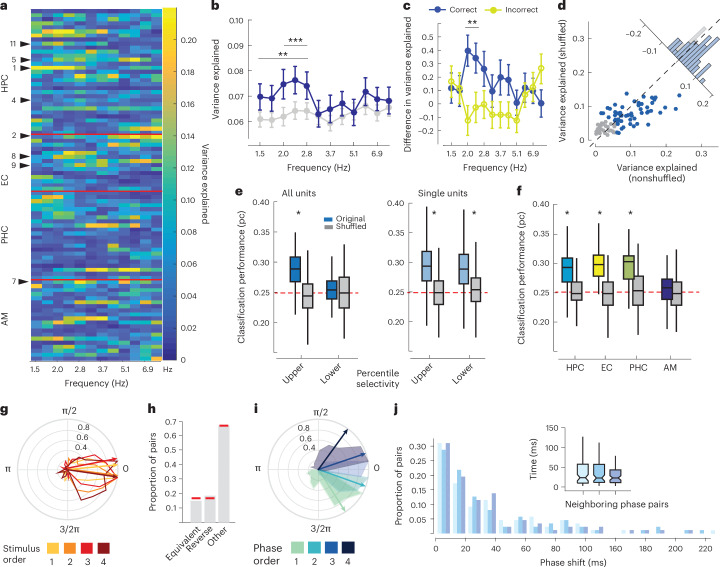
Preferred phase of firing varies with sequence position. **a**, The circular variance explained (*V*_ex_) between positions for units with high stimulus selectivity **b**, The average *V*_ex_ for nonshuffled (blue) and shuffled (gray) position labels. The error bars denote s.e.m. Stars indicate significance based on the permutation test (**P < 0.01 and ***P < 0.001, *N* = 1,999; one-sided, *N* = 87). **c**, The normalized difference in *V*_ex_ between nonshuffled and shuffled conditions for correct (blue) and incorrect (yellow) trials (*P* = 1.5 × 10^−3^ and 0.013, respectively, one-sided permutation test, *N* = 87). **d**, *V*_ex_ per unit shown for shuffled (*y* axis) versus nonshuffled position labels (*x* axis) at the theta frequency with the respective highest power increase during delay and corresponding histogram (inset; blue dots correspond to the upper 50th percentile across all units in either group). **e**, Position decoding performance based on preferred mean phase of firing during delay across the population of units (left) and individual units (right). A separate plot for highly versus less stimulus-selective units, and nonshuffled and shuffled position labels (median sign rank test, two-sided, individual units *P* = 2.6 × 10^−18^, *Z* = 8.7 for the higher selectivity group and *P* = 2.67 × 10^−18^, *Z* = 8.72 for the lower selectivity group, population: *P* = 6.86 × 10^−10^, *Z* = 6.17 and *P* > 0.05, *Z* = 0.4, for high (*N* = 87) and low selectivity groups (*N* = 96, respectively)). **f**, Population-based decoding performance for different MTL regions (median sign rank test, two-sided, *Z*_HPC_ = 6.7, *P* = 1.32 × 10^−11^; *Z*_AM_ = 2.2, *P* = 0.03; *Z*_PHC_ = 8.16; *P* = 3.37 × 10^−16^; *Z*_EC_ = 9.7, *P* = 1.82 × 10^−22^, *N* = 101 shuffles). **g**, Phase of spiking histograms and mean direction across units shown in **a** per stimulus position (color coded). Plotted are circular differences in phase with respect to mean phase across all positions per unit. **h**, The proportion of units for which the phase of firing order for different positions is equivalent to item order is reversed or is different. The red lines denote the proportion expected by chance (1/6, 1/6 and 5/6, respectively). **i**, An equivalent plot as in **g**, except here phases are assigned a ‘position’ label based on recorded phase order, not stimulus order. **j**, The distribution of three consecutive pairwise phase shifts in milliseconds based on neighboring phase groups shown in **g** as well as median pairwise time differences per neighboring pair (*N* = 87). The box plots in **e**, **f** and **j** are centered at the median and depict the interquartile range, and whiskers represent 1.5 times the interquartile range (Tukey box plot).

Next, we investigated theta phase differences between stimulus positions of a particular item (a neuron’s PS) during working memory as a function of theta frequency. We specifically targeted highly stimulus-selective neurons, aiming at identifying a clear link between encoding and maintaining a specific stimulus in memory. Overall, neurons exhibited phase differences between sequence positions in all investigated theta bands and MTL regions (Fig. 4a). *V*_ex_ was largest in the lower theta frequency range (2–3 Hz), in line with earlier findings investigating spike-phase coupling encoding stimulus identity^3^ (see also Fig. 4b). Critically, we found a similar effect choosing only the one theta frequency per unit–LFP pair for which we observed the highest oscillatory power increase from baseline to delay (paired *t*-test, *t* = 2.52, *P* < 0.02 across all units; Fig. 4d). This analysis not only accounts for variance in oscillatory peaks between LFP channels^10^ and the number of statistical comparisons when analyzing multiple frequencies, but also uses an independent criterion for selecting a specific theta frequency. We also repeated the analysis separately for groups of correct and incorrect trials to test whether the effect was related to task performance. Indeed, the normalized difference in *V*_ex_ (measured as the effect size Hedges’ *g* between nonshuffled and shuffled conditions) was significantly enhanced only for correct, but not for incorrect trials (permutation test, *P* < 0.05; Fig. 4c). Finally, we repeated all analyses for neurons exhibiting low stimulus selectivity. Here, phase separation between positions was not as apparent, in line with our reasoning that visual responsiveness to multiple items confounds a clear spike-phase relationship to a particular item under study (Supplementary Fig. 3).

Next, we tested whether sequence position can be decoded from phase of firing, employing the same SVM algorithm as described above. This analysis represents a different approach since it does not necessarily assume a linear relationship between phase and stimulus position (per neuron). For individual units, average decoding performance was better than chance (25%; Fig. 4e). We obtained similar results using population activity for highly stimulus-selective units, while this was not the case across the less stimulus-selective population. Additionally, decoding performance was similarly high in hippocampal, enthorinal and parahippocampal units, but substantially lower in the AM (Fig. 4f).

Finally, we investigated the relationship between the magnitude of spike-phase locking and encoding of position by phase. Similar to previous studies (for example, ref. ^11^), we observed that phase locking is enhanced during memory processing. At the same time, we found that different phases encode multiple stimulus positions during working-memory maintenance. To assess how these seemingly contradictory effects are related, we computed the rank correlation between mean kappa values derived from theta-related spiking across four stimulus positions versus variance explained *V*_ex_. Here, we observed a negative correlation between the two measures (Spearman’s rho −0.4183 and −0.5066, *P* < 10^−4^; Supplementary Fig. 4a,b, upper row). In contrast, when computing kappa separately for each position (and averaging afterward), we did not observe this correlation (Spearman’s rho −0.02 and −0.1895, *P* > 0.05; Supplementary Fig. 4a,b, lower rows). These results demonstrate that a more limited phase range (that is, higher spike-phase locking) is associated with worse encoding of stimulus positions. Interestingly, this effect appears to be mediated by larger phase separation between item positions rather than a higher kappa, that is, stronger phase locking per position and suggests a seeming tradeoff between phase locking and sequence coding. Future studies may investigate whether and how this relationship is influenced by other factors, for example, the number of items whose order needs to be remembered.

Taken together, several lines of analyses suggest that phase of firing indeed differs depending on the serial position of maintained memory items, as predicted by Lisman’s model, and that this is more clearly observed for neurons showing higher stimulus selectivity.

### Phase of firing order does not correspond to item order

Figure 4g,h summarizes our analyses regarding the central prediction of the Lisman model, namely a match between phase and item order within a sequence. We first show the phase distribution across neurons per stimulus position, normalized by subtracting the mean phase across all positions (Fig. 4g). Hence, if item position matched phase order, this would result in an equivalent ordering of phases across neurons. However, this was not the case. Similarly, when analyzing phase order for individual neurons using mean phase of firing per position, we found that approximately 15% of units exhibited the stimulus-equivalent consecutive phase order (circular ordering clockwise, that is, 1, 2, 3, 4), 18.4% of unit–channel pairs showed the reverse order (that is, 4, 3, 2, 1) with both proportions not significantly different from the expected chance probability of 1/6 for a specific order (chi-squared test of proportions, *P* > 0.05; Fig. 4h). Thus, while Lisman’s model proposes an equivalence between phase and stimulus order during working memory, this was not reflected in our empirical results. We also assessed the phase range used to encode item positions in memory. Figure 4i shows the mean phase distributions across neurons with phases sorted based on the preferred phase-of-firing order of each neuron (instead of actual item position). As expected from our spike-phase coupling results, the phase range representing all positions spanned only a fraction of the entire cycle (110°, interquartile range, 55.6°). Remarkably, within this range phase differences were equally distributed (median phase difference between neighboring-position pairs 27.7°, 27.4° and 24.9°, median time shifts 21.2 ms, 20.4 ms and 20.6 ms, Kruskal–Wallis test chi-squared of <0.1, *P* > 0.05; Fig. 4j). On the one hand, these findings support the notion that phase-coupled spiking might have a larger impact on target regions and foster effective communication and neural plasticity^14^. At the same time, equal phase differences provide an efficient way to maximize the representation of information in phase space, in our case four different item positions.

Taken together, our analyses reveal position-dependent phase-of-firing differences at the single-unit level during working memory. However, while our results support a phase-of-firing code for representing sequential items in memory, as suggested by the Lisman model, we find no equivalence regarding the ordering of spike phase and position. This seems difficult to reconcile with the theory, which clearly predicts a correspondence between phase of firing and stimulus order.

### Phase modulation in a trained RNN model

**Fig. 5:**
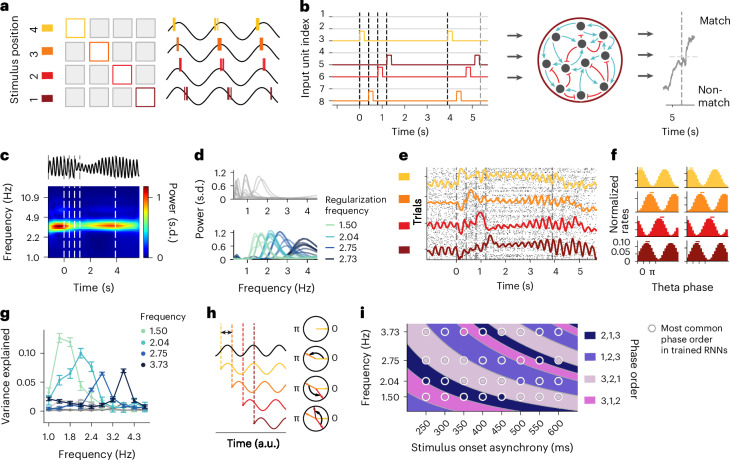
The oscillation phase of stimulus-selective units in trained RNNs varies with item position. **a**, A schematic Lisman and Idiart model. A stimulus-selective neuron spikes at different, consecutive theta phases in response to each of the four encoded sequential positions of a memory item (color coded). **b**, An RNN schematic on an example trial. The colored lines correspond to different stimulus inputs. **c**, An example LFP extracted from a trained network and averaged time–frequency spectrum of trained RNN activity showing theta power. **d**, The average power extracted from models without regularization (top) and with regularization (bottom). **e**, Raster plots (spikes sampled from rate activity) and mean rate (colored lines) of a stimulus-selective model neuron shows a clear evoked response at each stimulus position, as well as oscillatory activity during delay. **f**, Examples of phase histograms of recurrent units during delay show different peaks of activity related to stimulus position. **g**, The circular variance explained (*V*_ex_) between stimulus positions is elevated at tuned oscillation frequency (color coded, *N* = 26 ± s.e.m.) in comparison with shuffled position labels. **h**, A schematic showing stimulus-induced phase reset and the resulting phase order. The phase of firing is reset to a constant value at each stimulus position (colored lines), while the reference oscillation (black) is unaffected. Given an SOA and oscillation frequency, multiple stimuli cannot always be contained within one oscillatory cycle; hence, phase resets lead to specific phase order as a function of the timing of the stimuli with respect to the reference oscillation. **i**, The most common phase order for models (in circles) trained with different regularization frequencies and tested at different SOAs. The background color corresponds to the order predicted by the model and the same colors therefore indicate congruence between predicted and simulated phase orders. We consider circular permutations of the same phase order to be equivalent (for example, 1,2,3,4 = 4,1,2,3 = 3,4,1,2 = 2,3,4,1 is denoted as 1,2,3).

RNNs have previously been used to investigate neural computations during various cognitive tasks, including memory tasks^15–18^. We used RNNs to assess potential neural mechanisms underlying our findings by training rate-based RNNs on a task analogous to the one used during neural recordings. In brief, four out of eight input units were sequentially activated during each trial, mimicking the presentation of stimuli in the experiment. This was followed by a constant delay and a presentation of the four initial stimuli, in either a matching (‘correct’) or nonmatching (‘incorrect’) order (Fig. 5b).

We subsequently analyzed neural activity of the trained networks in a similar fashion as our neural data. First, we asked whether our models also exhibit oscillatory activity. We defined the model’s LFP (Fig. 5c) as the summed absolute synaptic input to all neurons^19^. We observed oscillatory power within our trained networks at a low-frequency range typically peaking between 0.4 and 2 Hz (Fig. 5d), with an increase in power during delay compared with baseline (Wilcoxon rank -sum test, *P* < 0.01). To make the models comparable to our empirical data, we used a novel regularization term to steer the frequency of the naturally occurring oscillations (Methods). For each of four frequencies (1.5, 2.04, 2.75 and 3.73 Hz), we trained ten models regularized to have oscillatory power at that frequency, of which 26 (6, 7, 5 and 8, respectively) models exhibited peak oscillatory power within 0.5 Hz of their regularization frequency (Fig. 5d, bottom). Next, we identified units with increased firing rate during stimulus presentation that were also selectively responding to a specific stimulus. Over all 40 regularized models, on average 155 ± 3.5 (mean ± s.e.m., *N* = 40) out of 200 trained units exhibited stimulus-responsive behavior (Wilcoxon signed-rank test, baseline versus stimulus presentation time, *P* < 0.001; example unit in Fig. 5e).

After demonstrating that RNNs exhibit both oscillations and stimulus selectivity, we investigated phase coupling in our model, focusing on the 26 models that successfully learned to oscillate at their regularization frequency. We created a model-based variant of spike-phase histograms by binning normalized firing rates above the 50th percentile of stimulus selective units during the delay with respect to a sinusoidal reference oscillation. Similar to our experimental data, the rate of the stimulus-selective units was coupled to the reference oscillation phase and the phase of peak rate differed between sequence positions (Fig. 5f). As in our empirical results, we quantified this effect by computing the circular variance explained (*V*_ex_) between item positions. We typically observed a significant increase in *V*_ex_ compared with shuffled position labels during the delay (Permutation Test, *P* < 0.05, *N* = 26; Fig. 5g). For the majority of these units (71.88%), phase order did not match item order within the sequence, similar to our neural recordings.

Our results demonstrate qualitative similarities between neural data and model activity and show order-dependent (but not order-preserving) phases in a neural network trained on a sequential memory task. Can our models help us understand how these nonordered phase relationships emerge? We noticed in our models that the theta oscillation of stimulus-selective units was systematically reset by the onset of their PS, and then remained at this phase during the delay period (Fig. 5e). In this case, the phase of such a unit, relative to an ongoing reference oscillation, is determined by the timing of the stimulus with respect to the reference oscillation. In other words, when successive stimuli are shown, the phase of firing to multiple stimuli at a specific stimulus onset asynchrony (SOA)—depending on the oscillation frequency—cannot always be contained within one oscillatory cycle. Thus, a phase reset leads to a specific phase order as a function of the timing of the stimuli with respect to the reference oscillation frequency (Fig. 5h). To test this hypothesis in our RNNs, we computed the phase orders of stimulus-selective units in models regularized to oscillate at one of four frequencies. For each model, we computed the phase order used for eight different SOAs (Fig. 5i). This gave us 11,593 units for which we predicted 56% of phase orders correctly (permutation test using shuffled frequencies and SOAs across units, *P* < 0.001; Supplementary Fig. 5c). In line with our hypothesis, we observed that different phase orders emerged in our models as a function of oscillation frequency and SOA (Fig. 5i).

Finally, we probed this relationship in our empirical neural data. For each stimulus-selective unit, we obtained the order of phase of firing at the theta frequency for which it exhibited the strongest phase differences between item positions (based on *V*_ex_). Subsequently, we quantified how many of these units exhibited a phase order as predicted by the frequency of the theta oscillation and the SOA used during the experiment. For a significant proportion of units, we were indeed able to predict the phase order correctly (25.2%, *N* = 87, permutation test using shuffled labels between frequency and ordering, *P* < 0.05). Taken together, our analyses link the phase order to the ratio between cycle duration of the oscillation and the SOA for both model and recorded neurons.

## Discussion

In this study, we tested a long-standing theory on the role of spiking and theta oscillations during sequence memory using single-unit and LFP recordings in human MTL as well as RNNs. We observed that spike rates did not vary with item position during memory maintenance. Instead, neurons exhibited robust spike-phase coupling to ongoing theta oscillation, where the preferred phase of individual neurons firing differed between item positions but did not reflect item order. Importantly, position encoding by spike phase depended on memory performance, emphasizing its behavioral relevance. When comparing neurons’ phase of firing at their local theta oscillation, we were not mixing different theta oscillations between neighboring electrodes. Nevertheless, whether phase shifts might also have resulted from micro-architectural differences (for example, CA1/CA3 within the HPC) cannot be answered with our recording approach.

RNN-based neural activity after training in an analogous task resembled many of our empirical findings. This included strong stimulus selectivity during encoding, emerging theta oscillations, phase coupling during memory maintenance and, importantly, phase differences related to item position, where phase-of-firing order again did not match the item order of the sequence. Why would sequence position be represented by phase of firing if the order is not preserved? A possible explanation is that sequence replay during memory does not necessarily reflect the (physical) stimulus order, but is altered, for example, through learning, thus rather reflecting internal processing states^20^. Similarly, MTL neurons do not stringently encode physical space or time^21^. Our findings are consistent with this notion: if there is an arbitrary—yet consistent—spike-phase relationship per neuron between different positions, then relational coding, as in reading out the position of stimulus A (from neuron X responding to stimulus A) versus the position of stimulus B (from neuron Y responding to stimulus B) would still be possible at the population level. Still, our results might contradict previous investigations on sequence memory using intracranial electroencephalography and magnetoencephalography that report theta-dependent gamma activity and corresponding item and phase order^22,23^. However, neither study explicitly required subjects to memorize temporal order nor did they measure SUA. Specifically, whether gamma activity indeed reflects selective responses to different visual stimuli during memory, such as those we have demonstrated for spiking neurons in the MTL, remains unresolved^24^.

How are our results related to short-term memory studies in rodents? Several studies report reliable, event-specific sequential firing of hippocampal assemblies in nonspatial memory tasks, where neurons’ sequential firing order did, however, not reflect their place field order^25,26^. To approximate sequential firing relative to item order in our data, we analyzed cross-correlograms for item-selective pairs of neurons during encoding and delay (Supplementary Figs. 7 and 8). While we observed that item order was reflected in equivalent time lags during encoding, this was not the case during delay, which might indicate independent rate and temporal coding in human sequence memory^27^. Moreover, in our model and data, oscillation frequency was relevant in predicting a particular phase order, whereas in rodents spiking of place cells relative to theta oscillations appears to stay constant at a range of theta frequencies^28^. Finally, Lisman’s original model assumes a shared theta oscillation across all neurons within one network. It is, however, unlikely for us to simultaneously record multiple visually selective neurons belonging to a shared theta rhythm due to the undersampling of neurons in our study and the known variability of theta oscillations throughout the MTL^29^. This limits the interpretation of relative phase offsets between neurons. Nevertheless, for a given neuron theta recorded at the same microwire is preserved across trials and Lisman’s model, equivalently, predicts theta-phase offset to increase monotonically with stimulus position, which we did not observe. Ultimately, a direct comparison between human and rodent studies will only be possible if high-density recordings of single-unit networks become available in human recordings.

Similar to our experimental findings, RNNs could maintain sequential information in working memory using ‘nonordered’ oscillatory phase of firing. Importantly, our models provide a potential mechanistic explanation for phase order through stimulus-induced phase reset, a phenomenon that has indeed been observed in MTL neurons^30–32^. Our modeling results are also in line with connectionist models implicating phase offsets of multiple oscillators in encoding serial position as well as oscillating neural networks that use a phase-of-firing code^33,34^. For RNN models, we provide a novel and alternative coding mechanism of working-memory content through emergent oscillatory dynamics in addition to known point, line and plane attractor dynamics^35,36^.

Taken together, our findings corroborate an important prediction of Lisman’s model—namely that the serial position of memory items is encoded in phase of firing, even if their order is not preserved. Thus, theta oscillations could indeed provide a temporal frame of reference for relating the ‘what’ to the ‘when’ as has been suggested previously^21^. Similarly, theta cycles could function as a ‘separator’ between sequential memory items to facilitate readout by downstream regions and thus subserve both efficient encoding and routing of information within working memory^14,37^. Ultimately, our observations point to a more general role of temporal coding based on oscillations within the MTL.

## Methods

Sixteen patients (nine female, seven male, median age of 42 and 45 years, respectively) with chronic, intractable epilepsy were implanted with depth electrodes to undergo seizure monitoring for presurgical evaluation. All subjects gave their written, informed consent to participate in the experiments. Subjects performed either 224 trials or 112 trials (one subject) of a modified Sternberg task as illustrated in Fig. 1a. Stimuli were displayed using Octave (https://gnu.org/octave) on a Debian 8 operating system (www.debian.org). On each trial, a fixation cross appeared for 1,000 ms. This was followed by a temporal sequence of four different stimuli that were chosen randomly on every trial out of a set of eight stimuli in total, during which subjects had to maintain fixation. Pictures were chosen based on a prescreening session 1–4 h before this experiment^38^. Pictures contained mostly natural images depicting photographs of people, places or objects. Within the sequence, each stimulus was presented for 200 ms with an interstimulus interval of 200 ms. The presentation of the fourth stimulus was followed by a delay period showing a blank black screen. The delay period ranged between 2,400 and 2,600 ms (median of 2,500 ms, interquartile range of 100 ms). Finally, a stimulus panel simultaneously showing four possible stimulus sequences was shown on the screen, one of which matched the sequence previously shown (chance level of 0.25). Subjects were instructed to press a key number indicating the row of the matching sequence. Each of the eight different stimuli used was presented equally often at each of the four temporal positions (for details, see the Supplementary Information). The recording techniques presented here have been described in detail in previous studies (for example, refs. ^38,39^). In brief, we recorded the raw voltage traces from nine microwires (eight high-impedance recording electrodes, one low-impedance reference; AdTech) protruding from the shaft of depth electrodes at a sampling rate of 32 kHz. Signals were amplified and recorded using a Neuralynx ATLAS system and referenced against one of the low-impedance reference electrodes. Data analysis was performed using custom-written functions as well as the CircStatsToolbox written for MATLAB version R2014b as well as the pycircstats toolbox written for Python^40,41^. Data figures were partially produced by the gramm plotting toolbox written for MATLAB^42^. Unless otherwise stated, we are showing Tukey box plots, that is, centered at median and depicting interquartile range, whiskers represent 1.5 times the interquartile range. Spike analyses: throughout the manuscript, we use the terms ‘neuron’, ‘unit’ and ‘cell’ equivalently to describe the recorded responses of presumed neuronal spiking. We use spiking equivalently to firing, to describe neuronal activity of single neurons. Model units describe the output activity of the artificial RNN units. Whenever multiple comparisons were performed, *P* values were corrected using the Simes procedure^43^. Spike sorting was performed semi-manually using Waveclus 2.0 and Combinato^44^. On the basis of thorough manual visual inspection of waveforms, we removed unit recordings that were contaminated by artifacts or were temporally unstable over the course of the recording, which resulted in a pseudo-population of 1,420 units from 921 unique LFP channels in MTL regions (HPC: *N* = 564, 376, 40%; EC: *N* = 251, 161, 18%; PHC: *N* = 213, 138, 15% and AM: *N* = 392, 246, 28%, units and channels, respectively). To assess whether a unit significantly responded to a stimulus, we compared spiking activity during prestimulus baseline (−500 ms before the first stimulus) with the stimulus period, and a unit was defined as responsive if there was a significant increase in spike rate relative to baseline (*P* < 0.001; for details see the Supplementary Information). We identified *N* = 217 highly stimulus-responsive units (*N* = 84 hippocampal units, *N* = 55 AM units, *N* = 55 parahippocampal units and *N* = 23 entorhinal units). The stimulus eliciting the largest firing rate increase relative to baseline is the PS and we compared PS versus NPS trials (where the PS had not been part of the sequence during encoding) to compare stimulus-specific effects. Since neurons could be responsive to multiple items, we separated two groups of neurons (‘high’ selectivity versus ‘low’ selectivity) based on a median split of the normalized difference in activity between the PS and the stimulus that elicited the second largest response (for details see the Supplementary Information). LFP analysis and spike-phase coupling: spectral analyses were similar to a previous report^37^. In brief, we obtained the time–frequency decomposition of the downsampled (1,000 Hz) LFP signal using complex Morlet wavelets (*c* = 7 wavelet oscillations) and extracted from the analytical signal the instantaneous amplitude and phase as a function of time and frequency. Changes in power during the delay were assessed by transforming the raw power spectra to the *Z*-score scale relative to baseline power. Delay spectra, as shown in Fig. 2b, were obtained by averaging across 1,500 ms before probe onset (equal to spiking activity window). For the spike-phase analyses, we focused on theta frequencies over a wide range of frequencies starting with 1.5, 1.75, 2.03, 2.37, 2.8, 3.2, 3.7, 4.4, 5.1, 5.9, 6.9 and 8 Hz. We analyzed simultaneously recorded spiking and LFP from the same microwire for 217 channel–unit pairs with 175 unique LFP channels. We used a baseline period of 1,000 ms and a delay period of 2,000 ms for comparisons (choosing the last 2 s before probe onset and leaving a minimum of 500 ms interval post stimulus offset). Circular analyses used Rayleigh’s test of uniformity and estimated the magnitude of spike-phase coupling using the concentration parameter kappa based on von Mises function fits to spike distributions across phase bins (for details, see the Supplementary Information). We performed several control analyses to test and account for possible spike-rate differences between conditions (Supplementary Information). To analyze whether the preferred phase of firing during the delay differed from stimulus positions during encoding, we obtained trial-based estimates of the mean preferred phase of spiking during the delay for each neuron during trials showing the PS of that unit within the sequence. We subsequently computed the circular variance explained between different experimental conditions (in our case stimulus positions 1–4) by quantifying the ratio of variance within conditions relative to the variance across conditions. We chose this nonparametric measure as it does not necessarily assume an underlying von Mises distribution. We defined circular variance within condition (that is, position) as ${V}_{j}^{\,w}=1-\left|\frac{1}{{N}_{j}}{\sum}_{k=1}^{{N}_{j}}{e}^{i{\theta }_{k}}\right|,$ where *N*_*j*_ is the number of trials in condition *j*, the index *k* runs across all trials of condition *j* and *θ*_*k*_ is the mean phase of spiking in trial *k*. We subsequently calculated the mean variance within conditions as ${V}^{\,w}=\frac{1}{N}{\sum }_{j=1}^{4}{N}_{j}{V}_{j}^{\,w}$, where *N* is the total number of trials and *N*_*j*_ the number of trials within condition *j*. While there were typically 28 trials in each condition, an unequal number of trials could potentially arise from the fact that trials during which no spikes were detected did not contribute to the mean phase estimate (on average 10% of trials across neurons). We further defined circular variance across all conditions as ${V}^{a}=1-\left|\frac{1}{N}{\sum }_{k=1}^{N}{e}^{i{\theta }_{k}}\right|$ where *k* runs across all *N* trials. We finally computed the circular-variance explained per neuron as ${{V}}_{\mathrm{ex}}=1-\frac{{V}^{\,w}}{{V}^{a}}$. For nonparametric statistical comparisons, we also obtained a random distribution of *V*_ex_ by shuffling trial labels *N* = 1,999 times between different conditions based on random permutations. We repeated this procedure for every unit using all trials, and separately for correct and incorrect trials (including the respective shuffle-based random distributions to account for trial-count differences between these two conditions). We also obtained an estimate for the proportion of units showing a significantly larger *V*_ex_ compared with their shuffled distribution. A unit was defined as significant if the true *V*_ex_ exceeded the shuffled *V*_ex_ estimates in at least 95% of the cases (*P* < 0.05) for at least two frequencies. To obtain confidence intervals on proportion estimates, we created surrogate distributions of units using a bootstrap procedure (with replacement, as implemented by bootstrp in MATLAB 2014b, *N* = 1,999) comparing the units’ *V*_ex_ and respective shuffled distributions for each drawn sample (Supplementary Fig. 6e). To estimate phase order, we first subtracted the mean phase across positions from each position’s phase, thereby anchoring all phases relative to 0° phase (see also Fig. 4g). We subsequently sorted phases counter-clockwise in an ascending order and obtained the stimulus-position index for the sorted phases accordingly. A phase order was defined as equivalent to position order if the relative ordering along the circle matched the stimulus order (expected by chance in one-sixth of cases), and reverse if the reverse stimulus order was matched (likewise expected in one-sixth of cases). For decoding analysis to test whether stimulus position can also be decoded from phase of spiking, we used a SVM (radial basis function kernel, one versus one encoding scheme) algorithm as implemented in MATLAB 2014b. Here, phase of spiking served as the predictor matrix to predict one of four possible binary class labels (that is, stimulus position) (chance performance 25%). Data were randomly partitioned into training and test sets for cross-validation (85% and 15%, respectively). Decoding performance was statistically compared with performance after shuffling true class labels across trials, that is, randomly assigning position indices to trial phases (*N* = 101). In addition to single-unit decoding, we also created a pseudo-population of units by pooling all units across sessions and theta frequencies. To estimate the effect size of position decoding between regions, we calculated the difference between true performance in standard deviation units (Hedges’ *g*, as described above) and obtained confidence intervals based on bootstrapping with *N* = 1,999 repetitions.

### Recurrent network model

#### Model definition

Our models consist of recurrent networks of *N* firing-rate units

1 $${\bf{\uptau }}\circ\frac{{\mathrm{d}}{\mathbf{x}}\left(t\right)}{\mathrm{d}t}=-\mathbf{x}\left(t\right)+\mathbf{J}\phi \left(\mathbf{x}\left(t\right)\right)+\mathbf{I}\mathbf{u}\left(t\right)+\sqrt{2{\bf{\uptau}}\sigma_{\xi}^2}\circ\bf{\upxi},$$

where ${\bf{\uptau }}\in {{\mathbb{R}}}^{N},$ is a vector of time constants, ${\circ}$ indicates element-wise multiplication, ${\mathbf{x}}(t)\in {{\mathbb{R}}}^{N}$ denotes the current of each unit, $\phi$ is the element-wise (nonlinear) activation function, ${\mathbf{J}}\in \mathbb{R}^{N\times N}$ is the recurrent weight matrix specifying the connectivity between units in the network, ${\mathbf{I}}\in \mathbb{R}^{N\times N_{in}}$ is the input weight matrix specifying the connectivity from stimulus input to recurrent units and ${\bf{u}}(t)\in \mathbb{R}^{N_{in}}$ is the time-varying stimulus input. $\,{\bf{\upxi }}$ denotes *N* Gaussian noise processes with zero mean and unit variance, representing intrinsic network noise scaled by $\sigma_{\xi}$. An overview of all parameters can be seen in Supplementary Table 1.

We implemented biophysical constraints on the weight matrix J in line with previous work^45^. We allowed neurons to have either only excitatory or only inhibitory outgoing connections (Dale’s law; Supplementary Information).

#### Task

We adapted the task performed by the human participants such that it could be readily performed by an RNN, keeping the information that had to be maintained during the delay period (four stimuli and their order) identical. The input to the network at a particular time step, **u**(*t*), was always ${\boldsymbol{0}}$, except during the stimuli and probe periods. During these periods four out of eight stimuli were activated sequentially for 0.2 s by setting the corresponding entry in the vector **u**(*t*) to 1. The activated stimuli were randomly chosen (without replacement) with equal probability every trial. After a delay period, the same four stimuli as during the stimulus period were shown, but now either in a different order (randomly drawn) or in the same order, with equal probability.

#### Training procedure

During training we simulated equation (1), using the Euler method with step size ${\Delta }_{t}$, giving us at time step *n*

$$\mathbf{x}^{n+1}=\left(1-\mathbf{\upalpha}\right)\circ \mathbf{x}^{n}+\mathbf{\upalpha }\circ \left(\mathbf{J}\phi \left(\mathbf{x}^{n}\right)+{\mathbf{I}\bf{u}}^{n}\right)+\sqrt{2\mathbf{\upalpha }\sigma_{\xi}^2}\circ {\mathbf{\upepsilon}},$$

with ${\mathbf{\upepsilon }}{{\sim }}{\mathscr{N}}\left(0,{\bf{I}}\right)$ and ${\boldsymbol{\alpha }}$ is an *N*-dimensional vector whose *i*th element is $\frac{{\Delta}_t}{{\uptau}_i}$. The network’s output is described by a linear readout of the firing rates *y*^*n*^ *=* **w**^⊺^**x**^*n*^ with ${\mathbf{w}}{{\in }}{{\mathbb{R}}}^{N}$.

For the network to perform the task, it had to determine whether the order of the initial sequence of stimuli matched the sequence presented after the delay period. We defined a scalar $\hat{y}$ that was either 1 (match) or −1 (non-match) during the decision period.

We defined the loss of a single trial as

2 $${\mathcal{L}}{{=}}\frac{1}{\mathop{\sum }\nolimits_{n=1}^{T}{m}^{\,n}}\mathop{\sum }\limits_{n=1}^{T}{m}^{n}{\left({y}^{n}-{\hat{y}}^{n}\right)}^{2}+{\lambda }_{\mathrm{FR}}{\rm{re}}{{\rm{g}}}_{\mathrm{FR}}+{\lambda }_{\mathrm{osc}}{{\rm{reg}}}_{\mathrm{osc}},$$

where *T* is the number of discrete time steps in one trial, *m* is a mask that is 1 during the decision period, otherwise 0. Here the ${\rm{reg}}$ terms denotes regularizers applied with corresponding weights $\lambda$. We applied an L2 penalty on the rates to prevent implausible saturation of the activation function ${\rm{re}}{{\rm{g}}}_{\mathrm{FR}}=\frac{1}{{NT}}\,\mathop{\sum }\nolimits_{n=1}^{T}\mathop{\sum }\nolimits_{i=1}^{N}\phi {\left(x_{i}^{n}\right)}^{2}$ and additional regularization to control the model’s oscillation frequency (equation (3)).

We optimized parameters of the network by minimizing equation (2) using gradient descent until 95% accuracy was reached on a validation set (details in the Supplementary Information).

#### Computing LFPs in RNNs

LFPs as recorded from the brain are generated from currents of neurons embedded in a three-dimensional space, where the exact arrangement of neurons hugely influences the recorded signal. Neurons in our model, however, are completely agnostic to physical space. Mazzoni^19^ compared various LFP proxies for standard leaky-integrate-and-fire (LIF) networks, and found that a specific linear combination of the LIF synaptic currents provides an accurate LFP proxy. However, the authors also suggested a simpler proxy that is plainly the (absolute) summed AMPA and GABA currents. Given that our units have even less detail then the LIF neurons, we calculated the LFP in line with this, by taking the summed absolute synaptic input as LFP

$${\mathrm{LFP}}^{n}=\mathop{\sum }\limits_{i=1}^{N}\mathop{\sum }\limits_{j=1}^{N}\left|\,{{{J}}}_{{ij}}\right|\phi\left({{{x}}}_{j}^{n}\right).$$

To systematically analyze the effect of oscillation frequency on the representations used by our model, we developed a new loss term to promote an LFP with a peak at a specified frequency during training. This allows one to shift the natural oscillation frequency of the model to a specific frequency. We applied this regularization to all models described in the main text.

For every training iteration we computed the LFP. To make the loss term amplitude-invariant, we first normalized the LFP thus ${\overline{\mathrm{LFP}}}^{\,n}=\frac{\mathrm{LFP}^{n}-{\mu }_{\mathrm{LFP}}}{{\sqrt{2}\sigma }_{\mathrm{LFP}}}$. We then took the norm of the Fourier component at a specified frequency

3 $${{\rm{reg}}}_{\mathrm{osc}}=-\left\|\frac{1}{T}\mathop{\sum }\limits_{n=1}^{T}{\overline{\mathrm{LFP}}}^{\,n}\exp -i2\uppi {f}_{\mathrm{osc}}n{\Delta }_{t}\right\|,$$

where ${f}_{\mathrm{osc}}$ is the regularization frequency in Hz. We selected four frequencies congruent with the range of frequencies found in our experimental data, and trained ten models for each frequency (1.5, 2.04, 2.75 and 3.73 Hz). Sixty-five percent of these models (26 out of 40; 6, 7, 5 and 8 for each frequency, respectively) exhibited peak oscillatory power within 0.5 Hz of their regularization frequency. For these models, the power at target frequency increased from 0.13 ± 0.022 to 0.68 ± 0.048 (mean ± s.e.m, *N* = 26), after training.

#### Model analysis

Analysis of the data output was performed in Python using the pycircstat package^41^ as well as custom-written code. For the subsequent analysis, we first generated 224 unique stimulus combinations matching the number of trials in the experiment. Half were randomly assigned to be match trials (the later four stimuli are presented in the same order as the initial stimuli) and the other half were assigned to be non-match trials (the order in which the initial and post-delay stimuli were presented differed). We repeated the analysis performed on the experimental data with the following adaptions to account for the model having a continuous firing rate instead of discrete spikes (Supplementary Information). For the rate-phase histograms (Fig. 5f) the reference oscillation was a sine wave, with a fixed phase at the onset of the last stimulus, and frequency matching the frequency with highest power in the models’ LFP spectra. For the analysis investigating frequency-related effects (Fig. 5g), we used only the 26 models that successfully learned to oscillate at their regularization frequency (peak oscillatory power within 0.5 Hz of their regularization frequency). For Fig. 5h, we analyzed an effect that is directly based on the oscillation frequency models used to encode stimuli, thus here we only included models with a *V*_ex_ at a frequency within 0.2 Hz of the applied regularization frequency (giving us *N* = 4.7 ± 1.2, mean ± s.d. models per SOA–frequency combination).

### Reporting summary

Further information on research design is available in the Nature Portfolio Reporting Summary linked to this article.

## Online content

Any methods, additional references, Nature Portfolio reporting summaries, source data, extended data, supplementary information, acknowledgements, peer review information; details of author contributions and competing interests; and statements of data and code availability are available at 10.1038/s41593-025-01893-7.

## Supplementary information

Supplementary Information Supplementary Figs. 1–8, Table 1 and Methods.

Reporting Summary

## Data Availability

Raw data from medical patients are not publicly available to protect patients’ privacy under the European General Data Protection Regulation. Data to reproduce the main figures and analysis of this study are publicly available via GitHub at https://github.com/mackelab/sequence_memory_NN.

## Code availability

Custom code for the RNN model as well as analysis code to reproduce main figures have been uploaded and are available via GitHub at https://github.com/mackelab/sequence-memory. Custom MATLAB code to reproduce main figures and analyses have been provided and uploaded and are available via GitHub at https://github.com/mackelab/sequence_memory_NN.
