# Supplementary material for: Phase of firing does not reflect temporal order in sequence memory of humans and recurrent neural networks
Source: Nat Neurosci. 2025 Mar 24;28(4):873–82. doi: 10.1038/s41593-025-01893-7 (PMC11976290; doi:10.1038/s41593-025-01893-7)
Supplement: Supplementary file 1 — Supplementary Figs. 1–8, Table 1 and Methods. [file 41593_2025_1893_MOESM1_ESM.pdf]

nature neuroscience

Supplementary information

https://doi.org/10.1038/s41593-025-01893-7

Phase of firing does not reflect temporal order in sequence memory of humans and recurrent neural networks

In the format provided by the authors and unedited

Supplementary Information

Supplementary Figures

S1

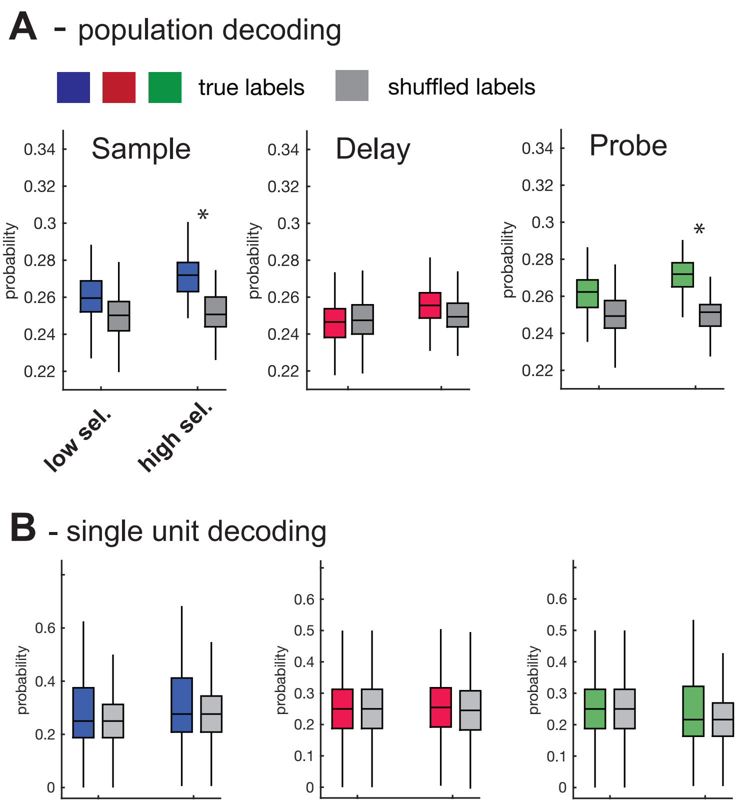

Figure S1. Decoding sequence position from spike rates. a. Population-based decoding of position for high and low stimulus-selectivity groups (N=87/96). Permutation test one-sided against shuffled position labels, $p<0.03$ for Sample/Probe for units showing high stimulus selectivity, $p>0.05$ for delay, N=199 shuffles. **b.** Same as a but single-unit-based decoding. Tukey Boxplots in a and b.

S2

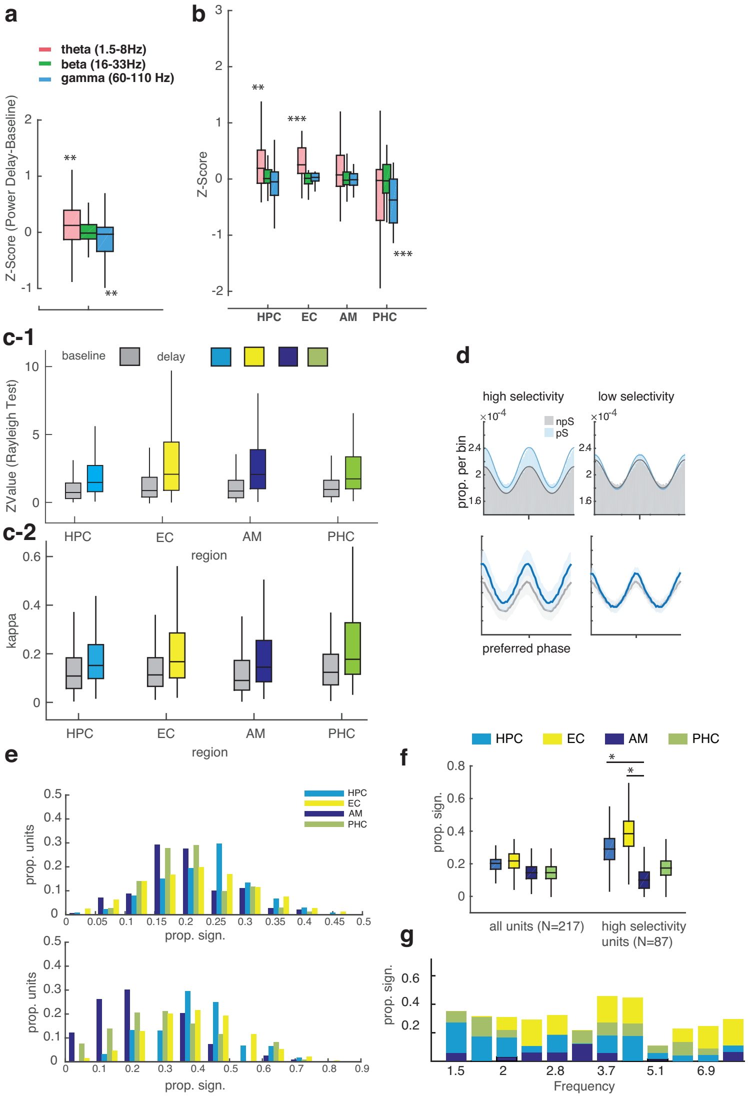

Figure S2. Additional spectral analyses. a. Normalized difference in oscillatory LFP power between baseline and delay (Z-score), showing significantly increased power in the theta band (including lower frequencies) and significant reduction in the gamma frequency band (60-110 Hz) during the delay (Wilcoxon signed-rank test, two-sided, p=0.008, N=178 unique LFP channels, based on visually responsive units) **b.** Same plot as in a, split by region. Mainly, significant theta power increases can be found in HPC and EC across all channels (Wilcoxon signed-rank test, two-sided, p<0.001, respectively, N=67/20/46/45 for HPC, EC, A and PHC, respectively) whereas A and PHC showed increases as well as decreases in theta power (p>0.05). Interestingly, significant gamma decreases during the delay can be only be found in PHC. **c.** Non-uniformity of theta-related phase of firing as indicated by Rayleigh's Z-score and kappa separately estimated from baseline and delay activity per region (Tukey boxplots). In all MTL regions investigated, we find significantly enhanced spike-phase coupling during delay compared to baseline (irrespective of PS/NPS, N=217 responsive units, 84 HPC/23 EC /55 A/55 PHC, Median signed-rank test, two-sided, baseline vs. delay, all regions p<0.001). **d.** Spike-phase histograms (upper panels) and van Mises fits (lower panels) estimated from theta band during delay for units with high vs. low stimulus selectivity, for trials containing the PS vs. not. Shaded areas correspond to SEM across units, N=87/96 for high- vs. low-selectivity units. Although the spike modulation seems to be larger for the high-selectivity group of units, estimates based on individual concentration parameters were not significantly different after correcting for spike rate differences. **e.** Histograms showing the distribution of proportions of units showing significant differences in phase-of-firing between stimulus positions based on Vex - Permutation tests per region. Histograms are shown for all units (upper panel, N=217) and again for units exhibiting high stimulus selectivity (lower panel, N=87). **f.** Proportion of units per region showing significantly elevated Vex compared to shuffled trials based on permutation tests (alpha=0.01), shown are median proportions across permutations. Among highly stimulus-selective

units, the highest proportions were found in HPC and EC (Median signrank test, two-sided, comparing proportions between regions, N=1999 shuffles, p<0.05). **g.** Same data as in **f.** per frequency and region, for stimulus-selective units (N=87). Tukey Boxplots shown in a, b, c and f.

S3

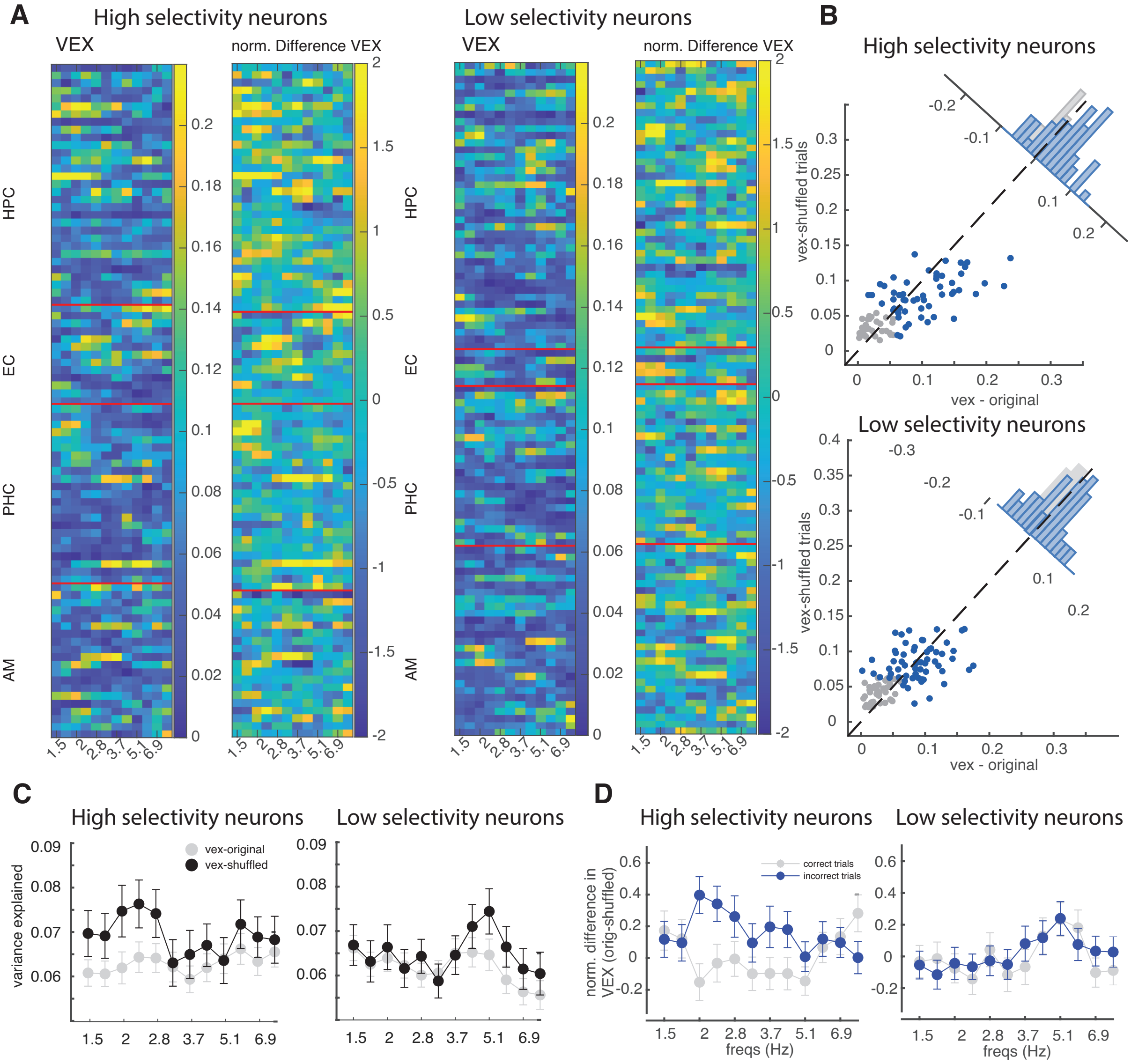

Figure S3. Phase-coding of stimulus position compared between high vs. low selectivity units. a. Comparison of absolute Vex (left) and normalized difference in Vex to the shuffled condition (effect size, right) for high- vs. low-selectivity neurons, sorted by region. Arrows indicate the example neurons shown in Fig. 4 of the study. For both groups, we find neurons whose phase is significantly different between stimulus positions based on a permutation test using Vex between the original and shuffled position labels. **b.** Comparison between groups of neurons when selecting the theta frequency with the highest power increase from baseline to delay, **c.** Averaged Vex for original and shuffled position labels as a function of frequency **d.** Normalized difference between original vs. shuffled position labels for correct vs. incorrect trials as a function of frequency. Error bars in c and d correspond to SEM, N=87/95 high- vs. low-selectivity neurons. Across the population, high-selectivity neurons show the effect of phase coding for position, whereas low-selectivity neurons do not. Plots for the high-selectivity group are the same as shown in Fig.4 of the manuscript.

S4

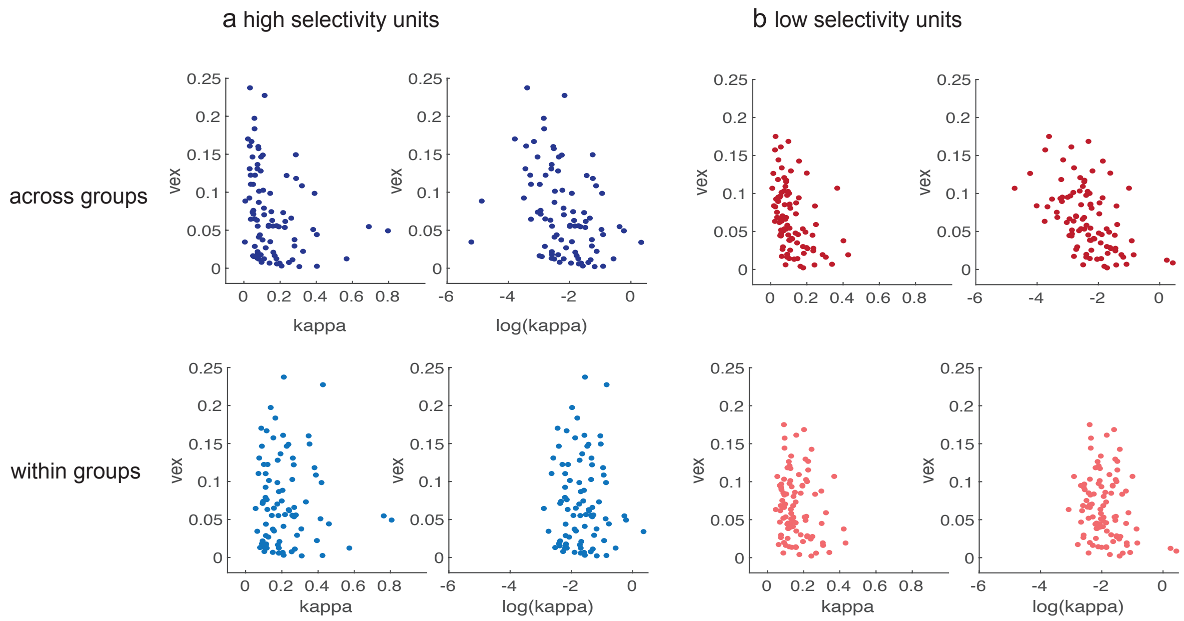

Figure S4 Relationship between spike-phase locking and variance explained. Phase-locking is negatively correlated with position encoding across stimulus positions, but not within position. For illustration purposes, data are plotted both on linear scale (left) and log-linear scale (right). **Top row:** Computing kappa across all trials yields a negative correlation between kappa and Vex for neurons with both high and low stimulus selectivity, which indicates that stronger overall spike-phase locking is associated with worse encoding of stimulus position by phase (Spearman's rho −0.4183 and −0.5066; p<10−4 for high- and low-selectivity units, respectively). **Bottom row:** Such a negative correlation was not found when kappa was estimated for individual positions (Spearman's rho −0.02 and −0.1895, p>0.05).

S5

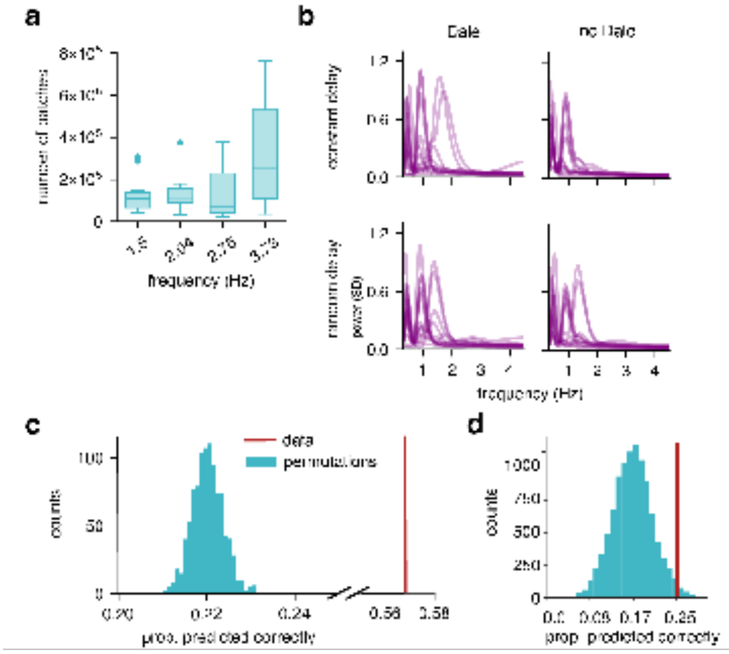

Figure 5S Model testing. a. Mean (±SEM) training duration of models (N=26) per regularization frequency. We noticed a frequency-dependent effect of training on convergence rate in line with existing

work [46]. **b.** Mean power during delay shows oscillations are ubiquitous in trained models, irrespective of the exact training setup. Here we trained 12 models per condition: With and without a randomized delay (rows; sampled from a uniform distribution with support [2400ms,2600ms)) and with and without enforcing Dale's law (columns; g=1, other parameters as in Supplementary Table T1). To accurately calculate power of potential low frequency oscillations, we extended the delay period to 10 s before calculating power. **c.** Permutation analysis for the model. We shuffle regularization frequencies and stimulus onset asynchrony (SOA) between units and calculate the proportion of phase orders predicted correctly. 56,4% of units exhibited the predicted phase order, significantly more than expected by chance (N=11593 units, permutation test, one-sided, using shuffled labels across frequency, SOA and phase order, $p<0.001$). The observation that the mean of the shuffled distribution is around 0.22 follows from the fact that our RNNs largely use the four phase orders we expect them to use based on our proposed interaction (Fig. 5i.; which would give a baseline of 1/4=0.25), but due to e.g. stochasticity in both the model simulations and training, the models end up using the other two phase orders for some of their units (thus shifting the baseline slightly towards 1/6). **d.** Permutation analysis for the data. We shuffle local field potential frequencies across recorded units and calculate the amount of phase orders predicted correctly. 25,2% of units exhibited the predicted phase order, significantly more than expected by chance (N=87 units, permutation test, one-sided, using shuffled labels across frequency and ordering, $p<0.05$)

S6

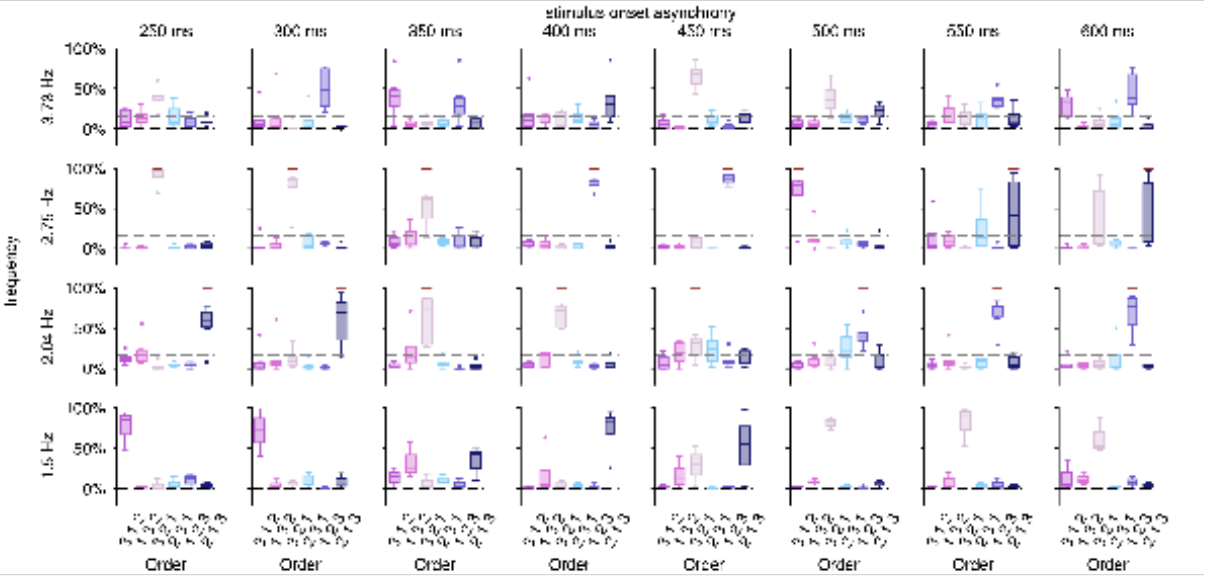

Figure S6 Relationship between SOA, oscillation and order in models (N=26). Raw data of Fig. 5i. We performed additional testing of our proposed interaction between stimulus onset asynchrony (SOA), oscillation frequency and phase order. In particular, we computed the phase orders used by models trained with four different regularization frequencies (rows), and tested at eight different SOAs (columns). Red bars denote the most common phase order predicted by our proposed interaction between SOA and oscillation frequency. Box plots indicate variation over different models. Since the phase-order predictions are based on oscillation frequency, we here included only models that learned to use oscillations corresponding to their regularization frequency; we took models with peak VEX at a frequency within 0.2 Hz of the applied regularization frequency (giving us N=4.7 ± 1.2, mean ± 1 std models per SOA-frequency combination). We can see that for almost all SOA-frequency combinations, the most common phase order found matches our prediction, for the four combinations where we are off, the second-most frequent phase order matches our proposed interaction. Note that we only compute 6 phase-orders per model, as we consider circular permutations of the same phase order equivalent (1,2,3,4 = 4,1,2,3 = 3,4,1,2 = 2,3,4,1 is denoted as 1,2,3).

S7

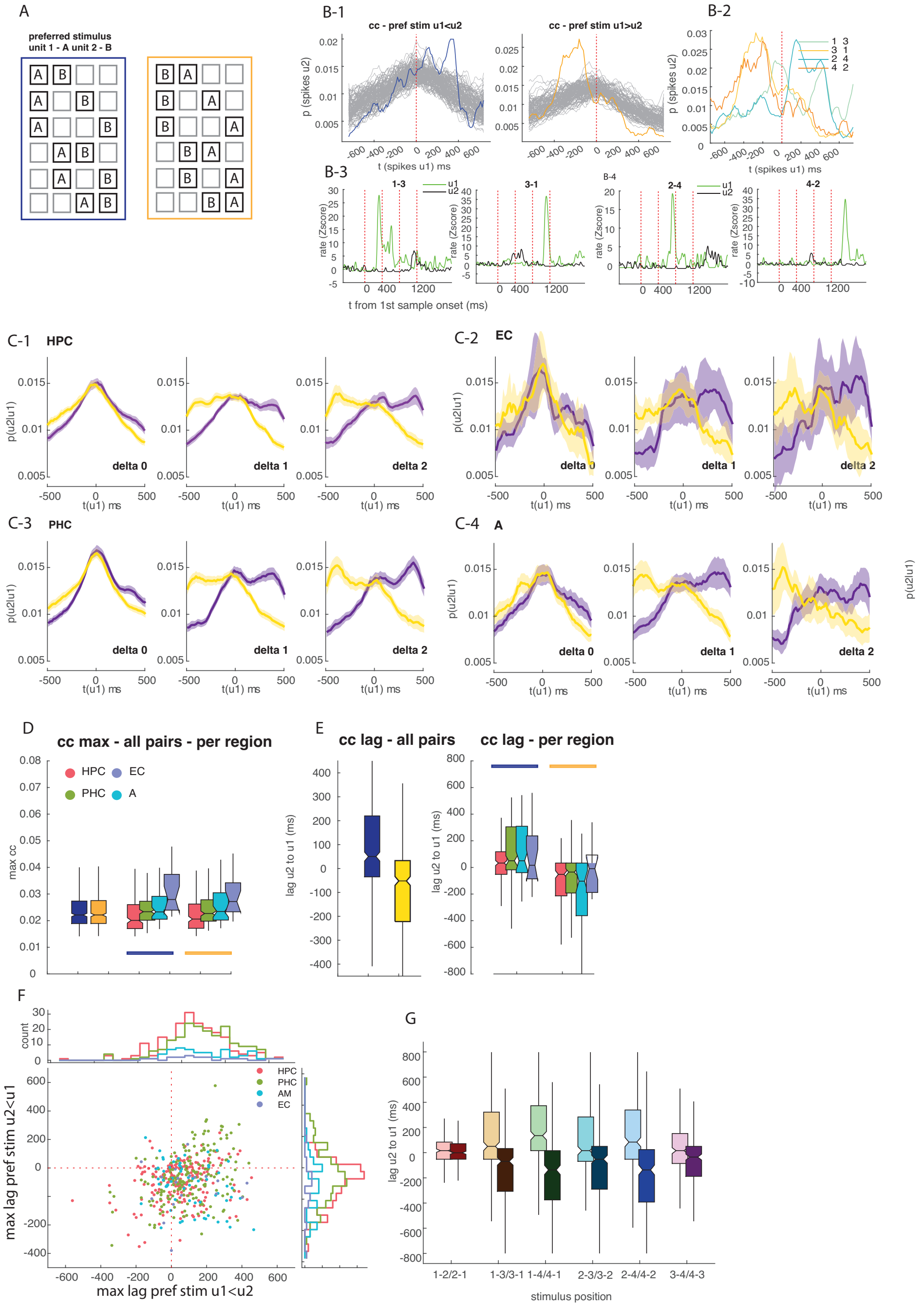

Figure S7 Cross-correlation (CC) analysis during encoding. a. Illustration of the two main experimental conditions for which CC was compared. Blue frame: trials for which preferred stimulus ('A') of unit 1 occurred before preferred stimulus ('B') of unit 2 and vice versa (yellow frame). In total, 6 position pairings can be compared separately (1-2-1, 1-3-1, 1-4-1, 2-3-2, 2-4-2 and 3-4-3). **b-1.** CCs (probability of firing of unit 2 at different time lags relative to unit 1) for one example unit. Blue lines depict CCs for trials during which the PS of unit 1 occurred before the PS of unit 2 (i.e., all pairings A before B), the yellow lines vice versa (i.e., B before A). Grey lines depict CCs derived from randomly shuffled trial pairings. **b-2.** Examples of CC for the two position pairings of this unit (labels indicate which position the stimulus was shown at), **b-3.** mean PSTHs of the activity of the two paired units for the example position pairings. **c1-4.** Average CCs ± SEM for different position pairings and all subregions. Delta 0: adjacent positions within a sequence (e.g. 1-2), Delta 1: one other stimulus in between, Delta 2: Two other stimuli in between. **d.** Median CCmax derived from cross correlograms per condition across all unit pairs (left) and separately per region (right). **e.** Median lags per stimulus order across regions (left) and per region (right) **f.** Distribution of time lags for the two conditions for all pairs color-coded per region. The majority of lags cluster in the lower right quadrant, indicating a positive lag for

trials when the PS of unit 1 occurred before the PS of unit 2 (i.e. A before B) and negative lags for the reverse order (i.e. B before A, McNemar test for non-independent proportions, Chi2=174.2, p=1e10−5). **g.** Median lags per position pairing. (samples in **d, e, g** for HPC: 204, EC: 18, PHC: 189, A: 61)

S8

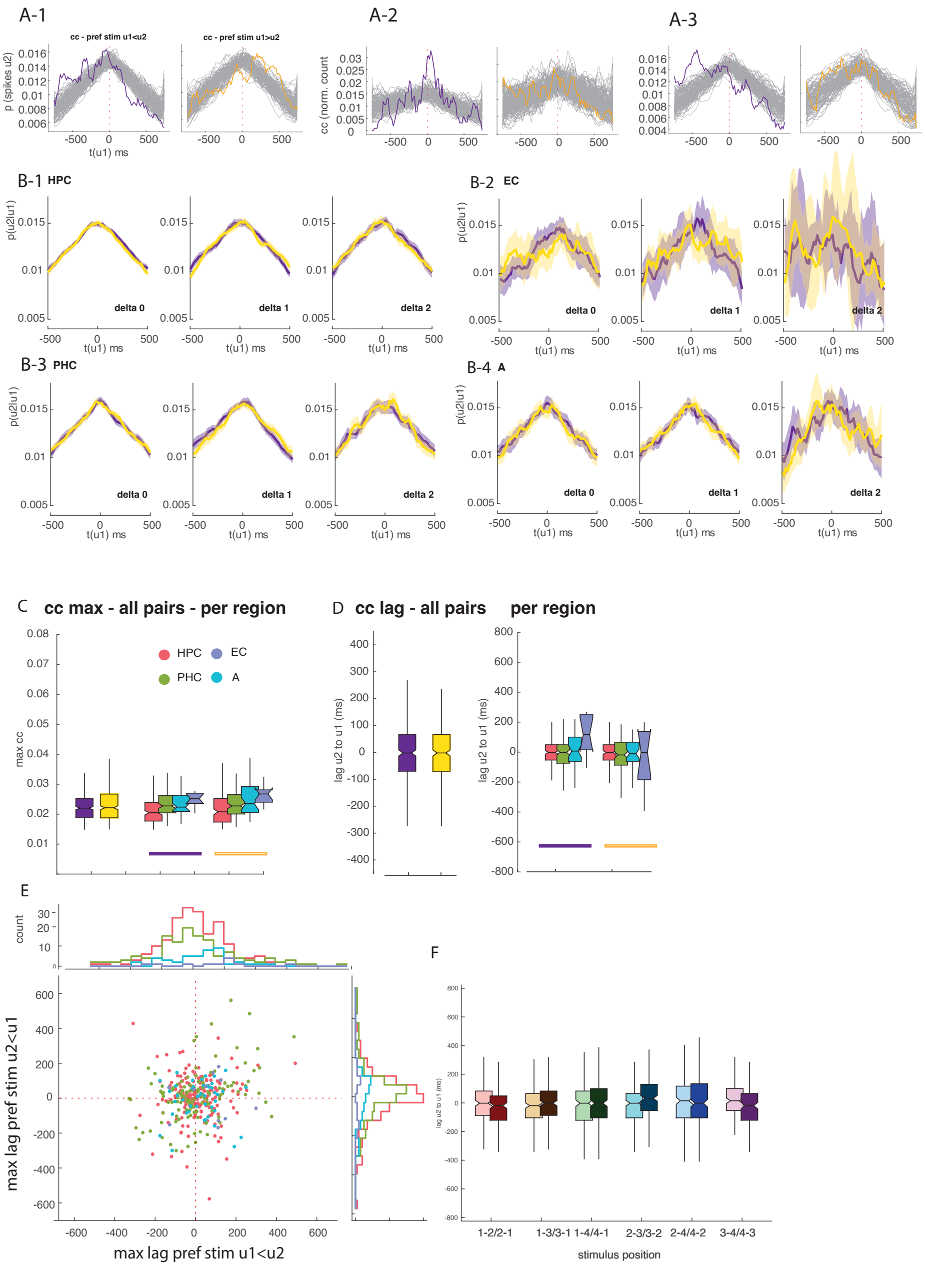

Figure S8 Cross-correlation (CC) analysis during delay (equivalent to Figure S6). a. Three examples of cross-correlograms for significantly correlated unit pairs for two order conditions. **b.** Averaged cross correlograms ± SEM per subregion for two order conditions. **c.** Maximum correlations across all pairs and per subregion **d.** Median time lags (ms) across all pairs and per subregion **e.** Distribution of time lags for the two conditions for all pairs color-coded per region. **f.** Median time lags (ms) per position-pairing. (Samples in **d, e, g** for HPC: 179, EC: 11, PHC: 129, A: 44 units were selected based on minimum number of 20 spikes during delay).

Tables

Key	Value
N	200
N_{in}	8
ϕ	tanh()
g	1.5
p_{inh}	0.2
σ_ξ	0.05
$\tau_{\min}$	20
$\tau_{\max}$	120
Δ_t	10
optimize	$\mathbf{I}, \mathbf{J}^{(\text{opt})}, \mathbf{w}, \boldsymbol{\tau}^{(\text{opt})}$
batch size	128
learning rate	5×10^{-5}
λ_{FR}	1×10^{-5}
λ_{osc}	1×10^{-1}

Table 1. Parameters used to train the RNNs

Supplementary Methods

Experimental Paradigm. Stimulus randomization: On each trial the sequence was randomized, such that subjects did not know the upcoming sequence on any given trial. In total patients completed 224 (112 for one subject) trials. Each stimulus was shown within the sequence on half of the trials (112 out of 224 trials) and not part of the sequence of images on the other half of the trials. Thus, we showed 112 (56 for one subject) trial unique sequences from a possible set of 1680 (8!/4!) within each experiment, where each image was shown an equal number of times at each position within the sequence (N=28 trials * 4 positions). The sequences shown had also the property that each pair of two images was shown exactly the same number of times (2 out of 8 is 28 possible pairs, each pair occurs four times, 4 * 28 = 112), and that each image was shown exactly the same number of times at each location (each image occurs 14 times at each location, 14 * 8 = 112 trials). The matching sequence in the probe display was shown counterbalanced in each row of the panel (i.e. each row approximately 25 percent). Within the probe sequences, images were counterbalanced in a way that the task could not successfully be solved (i.e. max. 50 % correct) when simply remembering the first, the last or the first two stimuli within the sequence. For each trial we obtained the keyboard response (i.e. sequence number 1-4) of the subjects and quantified the proportion of correct responses (PC) as the number of trials for which the subjects' response matched the previously shown sequence. Reaction times were computed as difference in time between probe onset and subjects' key press.

Spike analyses: We identified stimulus responsive units comparing baseline to 50ms bins after stimulus onset. For at least one 50ms window within the range of 250-600ms

post stimulus onset for hippocampal, entorhinal and amygdala units or 170-550ms post-stimulus onset for parahippocampal units there had to be a significant increase in response. By comparing activity during trials in which the PS was shown within the sequence (half of the trials, N=112) to trials in which it was not, we were able to track stimulus-specific effects in neural activity during the maintenance period. It could, however, still be the case that units sometimes responded to multiple stimuli. Thus, in order to relate spike-phase during the maintenance phase of the task (where no stimulus was shown) to the encoded position of *one particular* item, we also assessed selective units' responses to a particular stimulus relative to the other seven out of the eight stimuli shown. We reasoned that analyses related to preferred phase and encoded position were reasonable only if a neuron was highly selective to exactly one of the stimuli, since phase-of-firing of a neuron would only be related to maintaining a stimulus in memory for which the neuron is highly selective to. In contrast, this relationship could not be assumed the neuron was responsive to many different stimuli (as in case of low stimulus selectivity). Thus, for analyses specifically targeting the question of whether and how much theta phase differed between encoded stimulus positions of one stimulus (Fig. 4), we separated the group of responsive neurons into high- vs. low-selectivity neurons. Specifically, after selecting stimulus responsive units ($p<0.001$ during encoding, see above), we separated units (via median-split), based on the normalized spike-rate difference during the encoding period (Hedges' g, [47]) between the stimulus yielding the largest and the second largest response (again across all item positions). In summary, this selection process yielded 87 units for the high-selectivity group, and 96 units for the low-selectivity group. Assessing spike phase for one stimulus per encoded position relied on 28 trials (in contrast to 112 trials across all 4 positions). To achieve higher statistical robustness from larger spike counts and to be able to estimate spiking at lower theta frequencies, units also had to exhibit an average number of at least 2 spikes per trial within the delay period (85% of the units). Thus for each position at least 56 phase values contributed to the spike-phase calculation. For the unit shown in Fig. 1a the associated Hedges' g was 1.01, indicating that the response to the preferred stimulus was about 1SD larger than to the stimulus with the second highest response. The selectivity index of this unit fell within the upper 25th percentile of the distribution across the population. To estimate single unit activity across the entire trial period (Fig. 1) we binned spikes with a resolution of 1 ms and obtained instantaneous firing rates by convolving the spike trains with a Gaussian kernel (SD = 25 ms) per trial. We transformed instantaneous firing rates into Z-scores by normalizing to the mean activity and standard deviation during the 500 ms baseline period. To compare spiking between different trial windows, Z-scores were averaged during the visual response window (see above), the delay period (1500 ms window prior to probe onset) and after the probe onset (200-500 ms).

Spectral analyses. The raw real-valued time series x(t) was convolved with the complex Morlet wavelet w(t, f0) to obtain the complex output signal y(t, f0), also denoted as the analytic signal, where f0 denotes the desired center frequency of the wavelet function. The center frequencies f0 to obtain power spectra as shown in Fig. 2 were created by exponential spacing of 100 frequencies between $f=2^x$ with $x = 6/8,...,54/8$ resulting in a range of frequencies approximately between 1.5 and 108 Hz.

Spike-phase Coupling. We used Rayleigh's test of uniformity as a measure to assess how non-uniform the distribution of preferred phase angles is across the *population* of neurons within each MTL region at different task phases (see Fig. 2d). We also used Rayleigh's Z score as a test statistic to compare uniformity of spiking between different

regions. To compare mean preferred phase angle between regions, we estimated the population phase angle by averaging across mean phases of all neurons within each region. For each of the units we obtained spike phase histograms by binning spiking at theta phase into equally spaced phase bins with a width of 1/16 (as shown in Fig. 3). To account for non-uniformity of phases not associated with spiking, average spike-phase counts were normalized per bin by the number of occurrences of each particular phase bin during the considered trial window. We subsequently quantified the preferred phase angle (mean direction) as well as the magnitude of spike phase coupling (concentration parameter) by fitting von Mises density functions to the spike distribution across phase bins. This procedure was repeated across all trials during which the PS was shown vs. not (NPS trials) and separately for trials with different stimulus positions of the PS within the sequence (1-4). To achieve statistical robustness for spike-phase analyses units had to exhibit an average number of at least 2 spikes per trial within the delay period (85% of the units). Thus for each position at least 56 phase values contributed to the spike-phase calculation. Comparisons of spike-phase-coupling magnitude (i.e. kappa) can be confounded by differences in spike rates between compared conditions. Indeed, we observed small, yet significant differences in spike rates between baseline and delay activity across all units (median spike rate in Hz baseline , delay 2.8 , 3.6, IQR 4.7 , 5.5, Wilcoxon signed-rank test Z-Value 8.06, $p<10^{-5}$) as well as within different regions (Median spike rate in Hz Baseline, Delay HPC: 4.25, 4.67, EC: 1.81, 1.98, PHC: 2.82, 3.32, AM: 2.60, 2.83, IQR HPC: 7.46, 7.13, EC: 3.59, 5.49, PHC: 4.46, 5.47, AM: 3.24, 3.84; all units Wilcoxon signed-rank test Z=8.09, $p<10^{-15}$, for individual regions: HPC:Z=4.6152, EC:Z=2.29, PHC:Z=4.3726, AM:Z=4.3401, all p<0.02). To assess whether differences in spike-phase coupling could have resulted from the observed spike rate differences, we performed the following control analyses: We first computed the average spike rate (in Hz) for baseline and delay (see above) and subsequently split units into the upper and lower 50th percentile based on spike rate differences. As expected, for units in the lower 50% group, we found no significant difference in spike rates between windows ('Glptile', N=36, 11, 33, 27 for HPC, EC, PHC and AM, p>0.05 based on Wilcoxon signed-rank test). Comparing median values between baseline and delay for this group ('Glptile'), we still observed significantly increased spike-phase coupling during delay (Z>5.15, $p<10^{-6}$). In a second control analysis, we ranked units based on their difference in spiking activity between both windows and consecutively excluded units until we no longer observed a significant difference in spike rate ('Gexcl', N=51,20,48,39 for different regions, Wilcoxon signed-rank test p>0.05). Again for this group ('Gexcl') we observed significantly larger median during delay compared to baseline (Wilcoxon signed-rank test, Z>8.69, $p<10^{-16}$). We performed the same analyses controlling for differences in delay-related spiking between PS vs. NPS trials (Wilcoxon signed-rank test based on median spike rates during delay: N=217, Z=5.66, $p<10^{-7}$) and still observed a significant difference in spike-phase coupling between these two conditions (Wilcoxon signed-rank comparison median in 'Glptile' N=108, Z=6.55, 'Gexcl' N=7.89, $p<10^{-10}$). Finally, we also tested median values between the low vs. high selectivity groups of units (across all trials) after eliminating spike rate differences using the same procedure as described above (Median spike rate high vs. low, 4.52 and 3.6 Hz, Wilcoxon rank-sum test Z=-2.77, p<0.01). While we initially observed that high-selectivity neurons exhibit slight albeit significantly stronger spike phase coupling than the low-selectivity group (Wilcoxon rank-sum test comparing median Values Z=2.13, p<0.04), this effect was abolished after correcting for spike rate differences between the two groups (Wilcoxon rank-sum p>0.05, Z=1.86).

Additional details for RNN training and analysis

Parameterization. We constrained the recurrent weights of our networks to adhere to Dale's law. To achieve this, we initialized a matrix $\mathbf{J}^{(\mathrm{opt})}$ with samples drawn from a half-normal distribution: $\mathbf{J}_{ij}^{(\mathrm{opt})} \leftarrow |y_{ij}|$, $y_{ij} \sim \mathcal{N}(0, \frac{g^2}{N})$. The matrix $\mathbf{J}$ is then computed as the dot product of $\mathbf{J}^{(\mathrm{opt})}$ with a diagonal matrix $\mathbf{D}$ where the first $N(1 - p_{\mathrm{inh}})$ elements in $\mathbf{D}$ were set to a positive number, and the last Np_{inh} elements were set to a negative number. Here p_{inh} denotes the fraction of inhibitory neurons. We choose elements in $\mathbf{D}$ such that the expectation of the recurrent input to neurons stays 0 [45,48] and the average of the variance of the excitatory and inhibitory populations is $\frac{g^2}{N}$. Mathematically, this results in the bulk of the eigenspectrum of $\mathbf{J}$ lieing in a circle on the complex plane with radius g [48]. The resulting dynamics are then given by a sharp transition from stable to chaotic dynamics at $g = 1$ as $N \to \infty$ [49]. For finite networks (as used in our study), instead of a sharp transition, there is an intermediate region where one finds limit cycles. During training, we optimize $\mathbf{J}^{(\mathrm{opt})}$, and compute $\mathbf{J}$ as $|\mathbf{J}^{(\mathrm{opt})}|_{+}\mathbf{D}$; we rectify the elements in the $\mathbf{J}^{(\mathrm{opt})}$ by applying the ReLU function to ensure that the excitatory-inhibitory constraint is fulfilled.

To keep model time constants within a biologically plausible range $(\tau_{\mathrm{min}}, \tau_{\mathrm{max}})$, we applied an element-wise nonlinear projection map [50]. We chose $\boldsymbol{\tau}$ to be parameterized as

$$\boldsymbol{\tau} = \tau_{\mathrm{min}} + \sigma(\boldsymbol{\tau}^{(\mathrm{opt})})(\tau_{\mathrm{max}} - \tau_{\mathrm{min}}).$$

We initialized $\boldsymbol{\tau}^{(\mathrm{opt})}$ with samples drawn from $\mathcal{N}(0,1)$ and optimized $\boldsymbol{\tau}^{(\mathrm{opt})}$ during training. With σ being the logistic function, elements $\boldsymbol{\tau}_i$ of $\boldsymbol{\tau}$ approach τ_{max} as $\boldsymbol{\tau}_i^{(\mathrm{opt})} \to \infty$ and τ_{min} as $\boldsymbol{\tau}_i^{(\mathrm{opt})} \to -\infty$.

Additional optimization details. We optimized parameters of the network by minimizing Eq. 2 using back-propagation through time (BPTT), with the Adam [51] optimizer in TensorFlow [52] with default settings (first and second order moment equal to 0.9 and 0.999, respectively).

Training with BPTT notoriously suffers from exploding and decaying gradients [53]. In order to avoid the former we used gradient clipping. If the norm of the gradient $\|g\|$ was larger than some maximum g_{max}, we multiplied g with $\frac{g_{\mathrm{max}}}{\|g\|}$. To avoid vanishing gradients we employed curriculum learning and first train on a short delay (0.2s). Sweeps over multiple models were realized used the Weights & Biases toolbox [54].

Accounting for continuous rates during analysis. We account for having continuous rates instead of spikes during analysis as follows. To find stimulus-selective neurons, we used mean firing rates and counted activity starting with stimulus onset as stimulus-triggered activity. To create analogues to spike phase histograms, we binned continuous firing rate with respect to the phase of a reference oscillation, making sure that our bin size never exceeded our simulation time step. The reference consisted of a sine wave with frequency corresponding to highest power in the model's LFP spectrum. Units in our model used tanh, with range (-1,1) as activation

function, due to desirable properties with regards to propagation of gradients during training. During analysis the firing rates where first mapped to (0,1), using an affine map.

References

46. Susman, L., Mastrogiuseppe, F., Brenner, N. & Barak, O. Quality of internal representation shapes learning performance in feedback neural networks. *Phys. Rev. Res.* **3**, 013176 (2021).

47. Hentschke, H. & Stüttgen, M. C. Computation of measures of effect size for neuroscience data sets. *Eur. J. Neurosci.***34**, 1887–1894 (2011).

48. Rajan, K. & Abbott, L. F. Eigenvalue spectra of random matrices for neural networks. *Phys. Rev. Lett.* **97**, 188104 (2006).

49. Sompolinsky, H., Crisanti, A. & Sommers, H.-J. Chaos in random neural networks. *Phys. Rev. Lett.* **61**, 259–262 (1988).

50. Li, Y., Kim, R. & Sejnowski, T. J. Learning the synaptic and intrinsic membrane dynamics underlying working memory in spiking neural network models. *Neural Comput.* **33**, 3264–3287 (2021).

51. Kingma, D. P. & Ba, J. Adam: A method for stochastic optimization. In *3rd International Conference on Learning Representations* (2015).

52. Abadi, M. *et al.* TensorFlow: Large-scale machine learning on heterogeneous systems (2015). Available at https://www.tensorflow.org/.

53. Pascanu, R., Mikolov, T. & Bengio, Y. On the difficulty of training recurrent neural networks. In *International Conference on Machine Learning* 1310–1318 (2013).

54. Biewald, L. Experiment tracking with weights and biases (2020). Available at https://www.wandb.com/.
