# Supplementary material for: Phase of firing does not reflect temporal order in sequence memory of humans and recurrent neural networks
Source: Nat Neurosci. 2025 Mar 24;28(4):873–82. doi: 10.1038/s41593-025-01893-7 (PMC11976290; doi:10.1038/s41593-025-01893-7)
Supplement: Supplementary file 2 — Reporting Summary [file 41593_2025_1893_MOESM2_ESM.pdf]

nature portfolio

Corresponding author(s): Stefanie Liebe, Florian Mormann

Last updated by author(s): Nov 27, 2024

Reporting Summary

Nature Portfolio wishes to improve the reproducibility of the work that we publish. This form provides structure for consistency and transparency in reporting. For further information on Nature Portfolio policies, see our Editorial Policies and the Editorial Policy Checklist.

Statistics

For all statistical analyses, confirm that the following items are present in the figure legend, table legend, main text, or Methods section.

n/a	Confirmed	
☐	☒	The exact sample size (*n*) for each experimental group/condition, given as a discrete number and unit of measurement
☐	☒	A statement on whether measurements were taken from distinct samples or whether the same sample was measured repeatedly
☐	☒	The statistical test(s) used AND whether they are one- or two-sided<br>*Only common tests should be described solely by name; describe more complex techniques in the Methods section.*
☒	☐	A description of all covariates tested
☐	☒	A description of any assumptions or corrections, such as tests of normality and adjustment for multiple comparisons
☐	☒	A full description of the statistical parameters including central tendency (e.g. means) or other basic estimates (e.g. regression coefficient) AND variation (e.g. standard deviation) or associated estimates of uncertainty (e.g. confidence intervals)
☐	☒	For null hypothesis testing, the test statistic (e.g. *F*, *t*, *r*) with confidence intervals, effect sizes, degrees of freedom and *P* value noted<br>*Give P values as exact values whenever suitable.*
☒	☐	For Bayesian analysis, information on the choice of priors and Markov chain Monte Carlo settings
☒	☐	For hierarchical and complex designs, identification of the appropriate level for tests and full reporting of outcomes
☐	☒	Estimates of effect sizes (e.g. Cohen's *d*, Pearson's *r*), indicating how they were calculated

Our web collection on statistics for biologists contains articles on many of the points above.

Software and code

Policy information about availability of computer code

Data collection	Neurophysiological data were recorded using Behnke-Fried depth electrodes (AdTech, Racine, WI) equipped with microwire bundles protruding from the tip of the electrodes (3-5 mm). Data were amplified and recorded using a 256-channel ATLAS amplifier (Neuralynx, Bozeman, MT) and the Pegasus software (version 2.1.1, Neuralynx, Bozeman, MT). Stimuli were displayed using Octave (https://gnu.org/octave) 3.x series, on a Debian 8 operating system (www.debian.org).
Data analysis	Spike extraction and sorting was performed using Combinato (no version, https://github.com/jniediek/combinato). Statistical analyses were conducted in MATLAB 2014b and 2022b, including the Statistics and Machine Learning Toolbox (The MathWorks, Natick, MA) and Python (tensorflow2015, Scipy 1.2), Matlab toolboxes for circular statistics (CircStatsToolbox, Philipp Berens (2024). Circular Statistics Toolbox https://www.mathworks.com/matlabcentral/fileexchange/10676-circular-statistics-toolbox-directional-statistics), MATLAB Central File Exchange and Python Toolbox PyCircStat (https://github.com/circstat/pycircstat), Gramm data visualization toolbox (https://www.mathworks.com/matlabcentral/fileexchange/54465-gramm-data-visualization-toolbox). Custom MATLAB code to reproduce the main figures and analysis of this study are publicly available on GitHub: https://github.com/mackelab/sequence_memory_NN. Custom Python code to reproduce modelling results and analysis are available on GitHub under : https://github.com/mackelab/sequence-memory

For manuscripts utilizing custom algorithms or software that are central to the research but not yet described in published literature, software must be made available to editors and reviewers. We strongly encourage code deposition in a community repository (e.g. GitHub). See the Nature Portfolio guidelines for submitting code & software for further information.

Data

Policy information about availability of data

All manuscripts must include a data availability statement. This statement should provide the following information, where applicable:

- Accession codes, unique identifiers, or web links for publicly available datasets
- A description of any restrictions on data availability
- For clinical datasets or third party data, please ensure that the statement adheres to our policy

• All code to establish and train the recurrent neural network model as well as analysis code to produce figure 5 are available under https://github.com/mackelab/sequence-memory

• Data to reproduce the main figures and analyses of this study are publicly available on GitHub: https://github.com/mackelab/sequence_memory_NN

• Raw data from medical patients are not publicly available to protect patients' privacy under the European General Data Protection Regulation

Research involving human participants, their data, or biological material

Policy information about studies with human participants or human data. See also policy information about sex, gender (identity/presentation), and sexual orientation and race, ethnicity and racism.

Reporting on sex and gender	Sex of participants: 9 female, 7 male, according to clinical reports. Gender was not assessed explicitly. Our study did not include gender-specific analyses.
Reporting on race, ethnicity, or other socially relevant groupings	Subjects were chosen based on clinical assessment only (recruitment) and no records of race or ethnicity were made. We do not expect for these factors to affect our results or play a role in our scientific questions.
Population characteristics	median age = 42 (female) /45 (male) years, other information on population characteristics was not assessed. In this study, no characteristics of the population were included as covariates.
Recruitment	The study represents a 'rare-opportunity' sample. Participants are medical patients with therapy - refractory temporal lobe epilepsy that were chosen by an interdisciplinary medical board at the Department of Epileptology, University Hospital Bonn, Germany (Neurologists, Neurosurgeons, Neuroradiologists) to be eligible of undergoing invasive intracranial EEG Monitoring for presurgical evaluation of epilepsy surgery using macro electrodes. The corresponding author(s) were not part of this decision. Patients were asked and gave their written consent to participate in the invasive iEEG recordings as well as the additional implantation of micro electrodes after being asked by the main author(s) to record single neuron activity and local field potentials and participate in the experiments. Patients were informed that they could withdraw from research at any time, and without any impact on their clinical care.<br>All patients gave informed written consent to participate in this study in accordance with the Medical Institutional Review Board of the University of Bonn, Germany. Only patients over the age of 18 years were recruited.
Ethics oversight	Ethics Oversight held and study approved by the Medical Institutional Review Board of the University of Bonn (accession number 095/10 for single-unit recordings in humans in general and 249/11 for the current paradigm in particular).<br>Adheration to the guidelines of the Declaration of Helsinki.

Note that full information on the approval of the study protocol must also be provided in the manuscript.

Field-specific reporting

Please select the one below that is the best fit for your research. If you are not sure, read the appropriate sections before making your selection.

☒ Life sciences ☐ Behavioural & social sciences ☐ Ecological, evolutionary & environmental sciences

For a reference copy of the document with all sections, see nature.com/documents/nr-reporting-summary-flat.pdf

Life sciences study design

All studies must disclose on these points even when the disclosure is negative.

Sample size	No a-priori sample-size calculation was performed. In our total sample, we recorded activity from 1420 units on 921 unique LFP channels in Our comprehensive dataset easily complies with or exceeds current standards in the field of human single unit recordings (e.g., Jamali et al., Nature, 2024; Qasim et al., Cell, 2021). However, how many patients were recruited to participate in this study was based on an approximate assessment of how many neurons we needed to make statistically sound conclusions. Here, the first goal was to gather a sufficiently large sample (N>100 neurons) for statistical robustness with high recording quality (good single unit spike isolation, free of artifact, robust /stable

spiking across the duration of the experiment) distributed approximately equally across all participants for higher validity/reliability of results. The second goal was to gain a sample of neurons (N>100) for which we would obtain robust responses to presented stimuli. Based on previous experience and in line with aforementioned studies, this is the case for about 15-20% of sampled neurons. The quality of data also highly depends on correct electrode placement by the neurosurgeon and the number of implanted electrodes (i.e. macroelectrodes) , which is not determined by the experimenters but medical personnel.

Data exclusions	No subjects were excluded from the study. As part of the preprocessing, single unit activity and LFPs were visually screened for artifacts and rejected when contaminated before analyses specific to the experiment. Further data selection was done for scientific reason and is described in the Methods section of the manuscript.
Replication	We reproduced several effects known from previous studies in the field that are prerequisites for our novel analyses (e.g. theta power increase or spike filed coupling during memory maintenance). For several analyses yielding crucial results / conclusions we performed equivalent, yet non-identical analyses, obtaining the same results (e.g. using a traditional measure of explained variance as well as a machine learning based decoding algorithms on preferred phase of firing). To enhance repilicability and robustness, analysis concepts, analysis code and results were discussed and agreed upon by multiple authors. Whenever possible, findings were replicated at the single subject level (for example behavioral analysis) and single neuron / single LFP channel level (for example visual responses to stimuli) as well as across the population.
Randomization	No randomization procedure was involved and all subjects performed the identical experimental protocol (except for visual stimuli chosen).
Blinding	Since the experimental paradigm did not involve a 'control' group, participants were equally aware and carrying out the identical conditions. Moreover, our experimental design did not involve manipulation or withholding knowledge about information as part of the study. Therefore, blinding does not apply.

Reporting for specific materials, systems and methods

We require information from authors about some types of materials, experimental systems and methods used in many studies. Here, indicate whether each material, system or method listed is relevant to your study. If you are not sure if a list item applies to your research, read the appropriate section before selecting a response.

Materials & experimental systems

n/a	Involved in the study
☒	☐ Antibodies
☒	☐ Eukaryotic cell lines
☒	☐ Palaeontology and archaeology
☒	☐ Animals and other organisms
☒	☐ Clinical data
☒	☐ Dual use research of concern
☒	☐ Plants

Methods

n/a	Involved in the study
☒	☐ ChIP-seq
☒	☐ Flow cytometry
☒	☐ MRI-based neuroimaging

Plants

Seed stocks	*Report on the source of all seed stocks or other plant material used. If applicable, state the seed stock centre and catalogue number. If plant specimens were collected from the field, describe the collection location, date and sampling procedures.*
Novel plant genotypes	*Describe the methods by which all novel plant genotypes were produced. This includes those generated by transgenic approaches, gene editing, chemical/radiation-based mutagenesis and hybridization. For transgenic lines, describe the transformation method, the number of independent lines analyzed and the generation upon which experiments were performed. For gene-edited lines, describe the editor used, the endogenous sequence targeted for editing, the targeting guide RNA sequence (if applicable) and how the editor was applied.*
Authentication	*Describe any authentication procedures for each seed stock used or novel genotype generated. Describe any experiments used to assess the effect of a mutation and, where applicable, how potential secondary effects (e.g. second site T-DNA insertions, mosiacism, off-target gene editing) were examined.*
